# Inhibition of phosphoenolpyruvate carboxykinase blocks lactate utilization and impairs tumor growth in colorectal cancer

**DOI:** 10.1186/s40170-019-0199-6

**Published:** 2019-08-01

**Authors:** Emily D. Montal, Kavita Bhalla, Ruby E. Dewi, Christian F. Ruiz, John A. Haley, Ashley E. Ropell, Chris Gordon, John D. Haley, Geoffrey D. Girnun

**Affiliations:** 1 Department of Pharmacological Sciences, Stony Brook University, 100 Nicolls Rd, Stony Brook, NY 11794 USA; 2 Department of Pathology, Stony Brook University School of Medicine, 100 Nicolls Rd, Stony Brook, NY 11794 USA; 3 Marlene and Stewart Greenebaum Cancer Center, University of Maryland School of Medicine, 22 S Greene St, Baltimore, MD 21201 USA; 4 Stanford University, 450 Serra Mall, Stanford, CA 94305 USA; 5 Department of Pathology, Stony Brook University, 101 Nicolls Rd, BST Level 9, Room 191, Stony Brook, NY 11794 USA

**Keywords:** Lactate, Cancer metabolism, PEPCK, Metabolic flexibility, TCA cycle

## Abstract

**Background:**

Metabolic reprogramming is a key feature of malignant cells. While glucose is one of the primary substrates for malignant cells, cancer cells also display a remarkable metabolic flexibility. Depending on nutrient availability and requirements, cancer cells will utilize alternative fuel sources to maintain the TCA cycle for bioenergetic and biosynthetic requirements. Lactate was typically viewed as a passive byproduct of cancer cells. However, studies now show that lactate is an important substrate for the TCA cycle in breast, lung, and pancreatic cancer.

**Methods:**

Metabolic analysis of colorectal cancer (CRC) cells was performed using a combination of bioenergetic analysis and ^13^C stable isotope tracing.

**Results:**

We show here that CRC cells use lactate to fuel the TCA cycle and promote growth especially under nutrient-deprived conditions. This was mediated in part by maintaining cellular bioenergetics. Therefore targeting the ability of cancer cells to utilize lactate via the TCA cycle would have a significant therapeutic benefit. Phosphoenolpyruvate carboxykinase (PEPCK) is an important cataplerotic enzyme that promotes TCA cycle activity in CRC cells. Treatment of CRC cells with low micromolar doses of a PEPCK inhibitor (PEPCKi) developed for diabetes decreased cell proliferation and utilization of lactate by the TCA cycle in vitro and in vivo. Mechanistically, we observed that the PEPCKi increased nutrient stress as determined by decreased cellular bioenergetics including decreased respiration, ATP levels, and increased AMPK activation. ^13^C stable isotope tracing showed that the PEPCKi decreased the incorporation of lactate into the TCA cycle.

**Conclusions:**

These studies highlight lactate as an important substrate for CRC and the use of PEPCKi as a therapeutic approach to target lactate utilization in CRC cells.

**Electronic supplementary material:**

The online version of this article (10.1186/s40170-019-0199-6) contains supplementary material, which is available to authorized users.

## Background

Cancer cells reprogram their metabolism in order to maintain their bioenergetic and biosynthetic needs [1–3]. Otto Warburg first described this phenomenon in 1924, observing that cancer cells preferentially convert glucose to lactate even in the presence of oxygen [4]. For many years, the prevailing view held that cancer cells had reduced mitochondrial function in the form of decreased tricarboxylic acid (TCA) cycle activity and oxidative phosphorylation (OxPhos) [5, 6]. However, over the past decade, it has become appreciated that cancer cells not only have functional mitochondria, but that OxPhos is required to maintain cancer cell proliferation and progression [7–9]. The TCA cycle is more than a metabolic hub to generate reducing equivalents to fuel the electron transport chain. By virtue of various cataplerotic and anaplerotic reactions, the TCA cycle also generates metabolic precursors for both bioenergetic and biosynthetic purposes, enabling cancer cells to use this pathway for bioenergetics as well macromolecular synthesis to support their highly proliferative state.

Glucose and glutamine are two of the main substrates used by cancer cells to fuel the TCA cycle [10–12]. However, it is now apparent that cancer cells use additional substrates based on nutrient availability, especially under the reduced nutrient conditions found in the tumor microenvironment (TME). While levels of glucose and glutamine in plasma are ~ 5 mM and 0.5–1 mM, respectively, their levels in the TME are much lower [13]. Therefore, cancer cells must rely on other substrates as nutrients sources. The concentration of lactate in the TME is ~ 5 to 10 mM [14–16]. Additionally, cancer cells are continuously making lactate as a result of aerobic glycolysis [4]. Although originally thought of as a byproduct of glycolysis, recent studies show that pancreatic, lung, and breast cancers utilize lactate as substrate for the TCA cycle in vitro and in vivo [14, 17–19]. Indeed, lactate was used preferentially over glucose. These studies highlight the importance of lactate as a substrate for the TCA cycle and raise significant interest in targeting lactate uptake and/or utilization by cancer cells.

Recently, we showed that the metabolic enzyme phosphoenolpyruvate carboxykinase (PEPCK; HUGO: PCK1) increases colon cancer cell growth in part by promoting the use of both glucose and glutamine by the TCA cycle [20]. The ability of PEPCK to regulate the TCA cycle and metabolizing lactate via the TCA cycle in other organs prompted us to determine if inhibition of PEPCK could be a therapeutic approach to target lactate utilization in colorectal cancer. We show that colorectal cancer (CRC) cells utilize lactate as a substrate for the TCA cycle and cell growth, especially when nutrient conditions are limiting. Importantly, we show that pharmacological inhibition of PEPCK decreases lactate utilization and subsequently tumor cell growth in vitro and in vivo. This is in part mediated by increased bioenergetic stress. These studies strongly suggest the use of small molecule PEPCK inhibitors as a potential cancer therapeutic approach to target the increased lactate utilization by tumors.

## Methods

### Cell culture and reagents

All cancer cell lines were obtained from American Type Culture Collection (ATCC) except the Moser colon cancer cell line, which were obtained as described previously [20]. All cell lines were cultured in Dulbecco’s modified Eagle’s medium (DMEM) with 10% fetal bovine serum unless otherwise indicated. Low-nutrient media was made with by addition of 2.5 mM glucose and 1 mM glutamine to DMEM lacking glucose and glutamine (GIBCO, A14430-01). Complete media consisted of DMEM, which already contained 25 mM glucose and 4 mM glutamine, supplemented with 10% fetal bovine serum. Low-nutrient media contained DMEM lacking glucose and glutamine, with 2.5 mM glucose and 1 mM glutamine added, and supplemented with 10% fetal bovine serum. When indicated medium was supplemented with 10 mM sodium-l-lactate (Sigma, 71718), medium pH was checked using pH strips prior to use to ensure there was no change. pMSCV and PEPCK HT29 and colo205 non-target control and validated shRNAs against human PEPCK cells were generated as previously described [20, 21] Phosphoenolpyruvate and oxaloacetate levels were determined using commercially available kits according to the manufacturer’s direction (Biovision, K365-100 and Sigma, MAK070-1KT). The PEPCK inhibitor, PEPCKi, was obtained from Axon Medchem (Axon, 1165) and dissolved in dimethyl sulfoxide (DMSO). DMSO was used in equal amounts as a vehicle control. For proliferation assays, 50,000–100,000 cells were seeded in triplicate and treated with indicated doses of the PEPCKi or vehicle for 3–5 days. Spheroids studies were completed in low adherent plates (Thermo, 174931).

### Stable isotope tracing

Cells were seeded into 60-mm dishes and allowed to grow overnight. ^13^C_5_-glutamine and ^13^C_3_-sodium lactate (> 98% purity and 99% isotope enrichment for each carbon position; Cambridge Isotope Labs, CLM-1822 and CLM-1579-0.5) were used as tracers since they provide excellent analysis of overall central carbon metabolism, in particular the TCA cycle [20]. Cells were incubated for 6–24 h with the ^13^C tracer. Briefly, following label treatment, culture medium was collected and cell pellets were harvested. Specific extractions and analysis were performed as previously described and below [20, 22]. For polar metabolites, cells were washed and then scraped into 50% methanol to water, snap frozen three times, spun down and supernatant isolated. The supernatant was dried down, methoximated, and derivitized as previously described [23]. Samples were analyzed by GCMS. Phosphoenolpyruvate (PEP) was monitored at m/z 369–372, pyruvate at m/z 174–177, lactate at m/z 219–222, citrate at m/z 465–471, malate at m/z 335–339, fumarate at m/z 245–249, and succinate at m/z 247–251. Fatty acids were saponified and converted to their methylated derivatives. Palmitate was monitored at m/z 270, myristate at m/z 242 by GC/MS. To determine incorporation ^13^C_3_ lactate into lipids, cells were incubated with 10 mM ^13^C_3_ lactate for 16 h. Lipids were extracted and processed as previously described [22]. GC/MS was performed as described above for palmitate and myristate. Data were analyzed using Mass Hunter and abundance corrected using ISOCOR.

### OCR and ECAR measurements

Basal extracellular acidification rate (ECAR) and oxygen consumption rate (OCR) measurements were made using a Seahorse Biosciences XF96 analyzer (Agilent). Briefly, cells were seeded on a 96-well assay plate and incubated low-nutrient medium supplemented with and without 10 mM sodium lactate overnight. Cells were analyzed using Seahorse assay base media with and without lactate (Agilent, 103575-100). Assay medium consisted of Seahorse Base DMEM with addition of 2.5 mM and 1 mM glutamine at pH 7.4. Basal respiration and ATP-coupled respiration, represented as OCR, were measured using a Mitochondrial Stress Test assay as per manufacturer’s instructions in Seahorse Base DMEM with 2.5 mM glucose and 1 mM glutamine with and without PEPCKi (Agilent, 103015-100). Data was normalized to cell number via CyQUANT assay measured by fluorescence and represented as a.u. (arbitrary units) (Thermo Fisher, C7026).

### ATP assay

Cellular ATP and ADP levels were quantified using a commercially available kit (Sigma MAK135). Briefly, cells were incubated overnight with indicated doses of PEPCKi in low glucose and glutamine conditions. The following day media was removed from cells, and ATP was measured as per manufacturer’s instructions.

### Flow cytometry

10^5^ Colo205’s cells were seeded in triplicate in a 6-well plate and incubated at 37° for 48 h. Cells were synchronized using a double thymidine block or serum starvation overnight and treated with indicated doses of PEPCKi for 24–48 h. Cells were harvested for analysis by either propidum iodide (PI) staining or Annexin V. For PI staining, cells were fixed in ice cold ethanol then stained with PI (Sigma, P4864) and analyzed by flow cytometry (FACSCalibur). Cells were stained with Annexin V and 7-aminoactinomycin D (7AAD) using a commercially available kit (BD Pharmingen, 559763) and analyzed by flow cytometry.

### Immunofluorescence

Colo205 cells were grown on a glass chamber slides and incubated at 37° for 24 h. Cells were treated with 10 μM PEPCKi in 2.5 mM glucose and 1 mM glutamine DMEM overnight. Slides were fixed with 4% formaldehyde and subjected to immunofluorescence staining. Slides were probed for Ki67 (1:500; abcam, ab15580-100) and visualized with an Alexa 488-conjugated secondary antibody (Molecular Probes, A11070). Slides were cover slipped, and images captured using an Olympus Fluoview FV1000 confocal microscope. Slides were observed using a × 60 Plan Apo N 1.42 oil immersion objective.

### Animal treatment studies

Animal experiments were performed according to procedures approved by the Stony Brook University IACUC. 2 × 10^6^ HT29 and Colo205 cells were inoculated into the flank of 6-week-old nude mice. Tumors were measured, and tumor size was determined as previously described [20]. Once tumors became palpable, mice were administered 10 mg/kg PEPCKi every other day and tumors measured for an additional 10 days.

### Western blotting

Cells and tissues were homogenized in RIPA buffer (EDM Millipore, 20-188) with phosphatase and protease inhibitor cocktails added. Proteins were separated on a 4–12% SDS polyacrylamide gel, transferred to nitrocellulose paper, and probed with antibodies against mouse and human PEPCK (Abnova, H00005105-M01) and PEPCK (Cayman Chemical, 10004943). Primary antibodies for AMP-activated protein kinase (AMPK; Cell Signaling, #2532), pAMPK (Cell Signaling, #2535), ACC (Cell Signaling, #3662), pACC (Cell Signaling, #3661), poly ADP ribose polymerase (PARP; Cell Signaling, #9532), and Actin (Sigma, A1978) were incubated at a 1:1000 dilution in 5% milk overnight, except actin which was 1:10,000 dilution. HRP-conjugated secondary antibodies (Jackson Immunoresearch, 715-035-150 and 715-035-152) were used and proteins visualized using enhanced chemiluminescence (ECL; Thermo Scientific, 32106).

## Results

### Colon cancer cells use lactate for cell growth

**Fig. 1:**
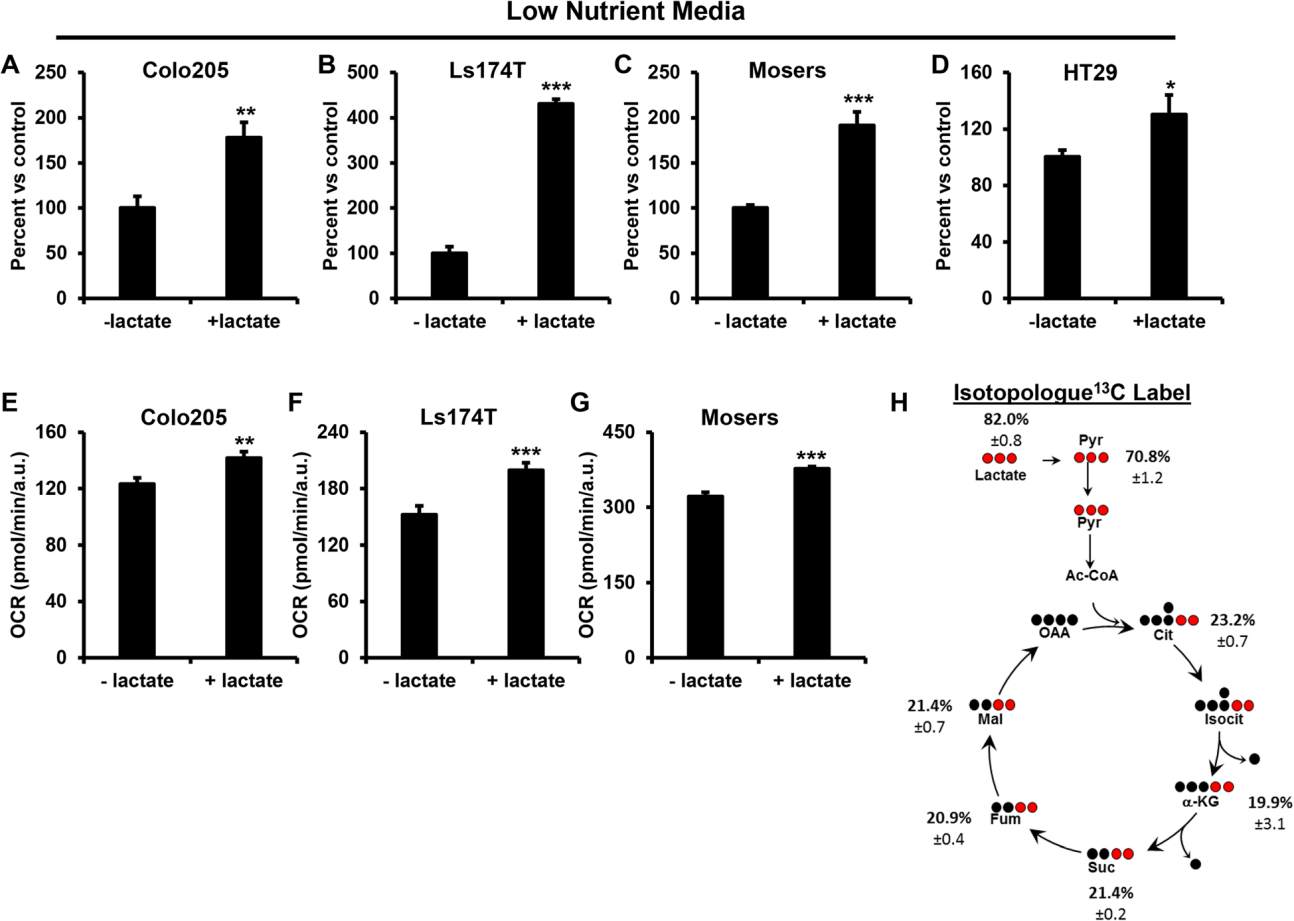
Lactate promotes cell growth and is metabolized via the TCA cycle in CRC cells. **a** Colo205, **b** Ls174T, **c** Moser, and **d** HT29 cells were cultured in reduced nutrient media with and without 10 mM lactate. Cell number was determined after 6 days using a Countess automated cell counter. *N* = 3 ± SD. **e** Colo205, **f** Ls174T, and **g** Moser cells were cultured in reduced nutrient media with and without 10 mM lactate and OCR determined using the XF^e^ Seahorse Bioanalyzer. *N* ≥ 15 ± SEM. **h** m + 2 enrichment of ^13^C from ^13^C_3_ lactate into TCA cycle metabolites in CRC cells. Colo205 cells were seeded in 6-cm dishes and incubated in reduced nutrient media overnight and then supplemented with 10 mM ^13^C_3_ lactate for 6 h. Metabolites were analyzed using GCMS. *N* ≥ 3 ± SD. *p < 0.05, **p < 0.01, ***p < 0.001

Recent studies show that lactate is a major contributor of carbons for the TCA cycle in breast, lung, and pancreatic cancer [17–19, 24, 25]. This prompted us to examine whether lactate is an important nutrient source in colorectal cancer (CRC) cells. Intratumoral lactate levels are reported to be in the range of 5–10 mM [14–16]. Therefore, cells were cultured in complete media (see the “Methods” section) in the absence and presence of 10 mM lactate. Culturing with lactate did not appear to alter cell proliferation in complete media (Additional file 1: Figure S1A–D). Tissue concentrations of many nutrients, in particular, glucose and glutamine, are much lower (5 mM and 0.5-0.8 mM respectively) than concentrations found in typical cell culture conditions. This is even more relevant with regard to tumors given the reduced nutrient levels in the tumor microenvironment (TME) (13). Therefore, we cultured cells in media with reduced glucose and glutamine (reduced nutrient media). The growth of the cells in this nutrient-deprived media led to a 65% reduction in cell number compared to complete media (Additional file 1: Figure S1E). Addition of lactate to cells under reduced nutrient conditions was able to rescue cell growth to varying degrees depending on the cell line (Fig. 1a–d). In order to determine if there was a dose-dependent effect, Colo205 cells were incubated in nutrient-deprived media with increasing amounts of lactate (Additional file 1: Figure S1F). Lactate increased cell growth in the Colo205s with increasing amounts of lactate.

### Colon cancer cells use lactate to fuel the TCA cycle

We then examined the utilization of lactate under these reduced nutrient conditions. We measured the effect of lactate on oxygen consumption (OCR) in order to determine if cells were oxidizing lactate via the TCA cycle and OxPhos. Lactate increased basal OCR by 15–30%, illustrating that the cells were using lactate for oxidative metabolism (Fig. 1e–g). Despite the addition of lactate to the media, we observed a decrease in extracellular acidification rate (ECAR) suggesting an increase in lactate uptake and utilization (Additional file 2: Figure S2A–C). Next, we investigated how colon cancer cells metabolize lactate using stable isotope tracer analysis. Cells were cultured with ^13^C_3_ lactate, and incorporation of ^13^C into pyruvate and TCA cycle intermediates determined. Over 80% of the total pyruvate fraction was ^13^C labeled (Σm1…m3), hence derived from lactate, i.e., not glucose (Additional file 2: Figure S2D). In addition, we observed ~ 20% m + 2 enrichment of ^13^C from lactate into TCA cycle intermediates citrate, α-ketoglutarate, succinate, fumarate, and malate indicating PDH mediated entry of lactate carbons into the TCA cycle via pyruvate (Fig. 1h). We also observe ~ 50–70% of total labeled TCA cycle intermediates (Additional file 2: Figure S2D). This effect was not just a result of the reduced glucose, since even when cultured in super-physiological concentrations of glucose (30 mM), m + 2 enrichment of ^13^C from lactate into these TCA cycle intermediates was ~ 13–22% (Additional file 2: Figure S2E). These data demonstrate that colon cancer cells metabolize lactate in part via the TCA cycle.

### Targeting lactate utilization via PEPCK

**Fig. 2:**
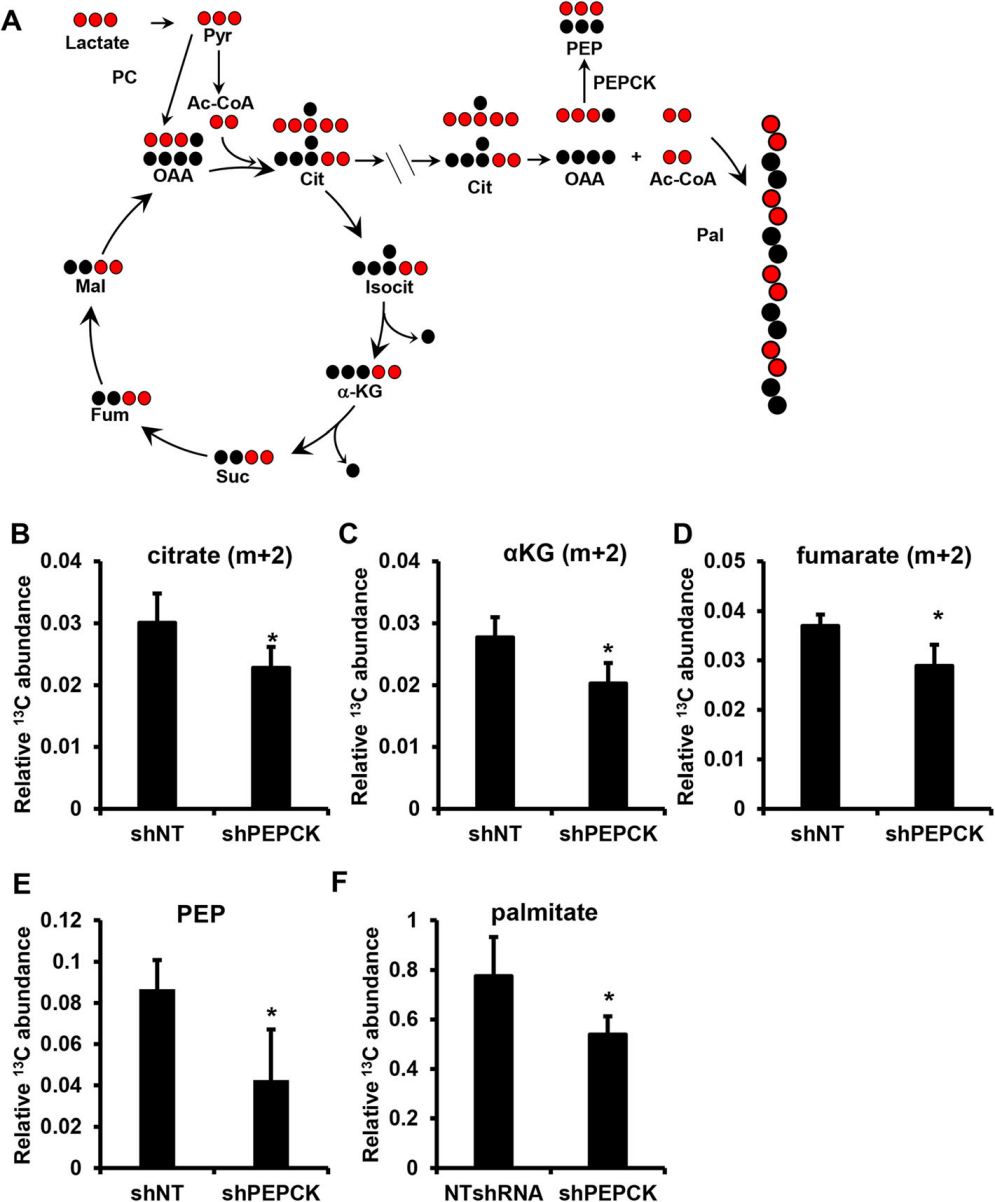
PEPCK promotes lactate utilization. **a** Schematic of ^13^C_3_ lactate incorporation into various metabolites. Relative abundance of m + 2 **b** citrate, **c** αKG, **d** fumarate, and **e** total ^13^C PEP were determined in shNT or shPEPCK Colo205 cells following incubation with ^13^C_3_ lactate. **f** Relative abundance of total ^13^C palmitate in shNT or shPEPCK Colo205 cells following incubation with ^13^C_3_ lactate. Cells were seeded in 6-cm dishes and incubated in reduced nutrient media overnight with and without 10 mM ^13^C lactate for 6 h (polar) or 16 h (non-polar), and metabolites harvested and analyzed using GC/MS *N* ≥ 3 ± SD. *p < 0.05, **p < 0.01, ***p < 0.001

There have been efforts to target lactate production since its discovery as a key metabolite in tumor metabolism [25–27]. However, translating these studies to the clinic has not been successful. Therefore, we sought to target the ability of cells to metabolize lactate by focusing on its main point of utilization, the TCA cycle. Several years ago, we showed that PEPCK promotes the utilization of glucose and glutamine via the TCA cycle in colon-derived cancer cells [20]. Therefore, we wanted to determine whether PEPCK altered lactate utilization in CRC cells. We used PEPCK knockdown cell lines that we had previously characterized and cultured under low-nutrient conditions in the presence of ^13^C_3_ lactate (Fig. 2a and Additional file 3: Figure S3A) [20]. Knockdown of PEPCK reduced the incorporation of ^13^C from lactate into TCA cycle intermediates (Fig. 2b–d). Importantly, the product of PEPCK, PEP, was significantly reduced (Fig. 2e). This demonstrates that the ability of CRC cells to utilize lactate is in part controlled by PEPCK. Interestingly, we do not observe an increase in intracellular ^13^C_3_ lactate with knockdown of PEPCK (Additional file 3: Figure S3B).

We previously showed that PEPCK promotes the incorporation of glucose and glutamine carbons into lipids [20]. Therefore, we wanted to determine whether lactate was also a substrate for lipid synthesis. Lactate is converted to pyruvate, which is then oxidized to acetyl coenzyme A (CoA) in the mitochondria, condensing with oxaloacetate (OAA) to form citrate (see Fig. 2a). Citrate can either be metabolized via the TCA cycle or leave the mitochondria and be converted back to OAA and acetyl CoA, where the acetyl CoA can be used for lipid synthesis. We examined whether lactate was incorporated in palmitate, the first product of de novo fatty acid synthesis. We observed 10% enrichment of ^13^C into palmitate after 16 h (Additional file 3: Figure S3C). We then carried out a similar experiment in non-target and shPEPCK cells. Loss of PEPCK reduced the enrichment of ^13^C into palmitate by ~ 30% (Fig. 2f). This demonstrates that cells are not only using lactate as a TCA cycle substrate but also as biosynthetic substrate, a phenomenon not previously described. Furthermore, these data show that PEPCK promotes lactate utilization for oxidative and anabolic metabolism.

### Pharmacologic inhibition of PEPCK

Our data using genetic loss of function experiments shows that PEPCK promotes lactate utilization. However, a pharmacological approach is required to target PEPCK therapeutically. PEPCK inhibitors were previously developed as antidiabetic agents [28–30]. We used a commercially available inhibitor that shows efficacy in the low micromolar range (Additional file 4: Figure S4A) [31, 32]. Previous studies showed that this inhibitor can decrease gluconeogenesis (GNG) in the liver. We wanted to determine whether the PEPCKi could inhibit PEPCK and the TCA cycle in colon cancer-derived cells, which do not undergo GNG [20, 31, 32]. Colo205 cells (which express abundant levels of endogenous PEPCK) were cultured with the PEPCKi, and a biochemical assay for PEP and OAA was performed. PEP, the product of PEPCK, was decreased, and OAA, the substrate, increased (Additional file 4: Figure S4B, C). Glutamine is an important substrate for the TCA cycle. Previously, we showed that loss of PEPCK decreased glutaminolysis and PEP production [20]. Therefore, we cultured Colo205 cells with ^13^C_5_ glutamine and determined the effect of the PEPCKi on glutamine utilization through the TCA cycle (Additional file 4: Figure S4D). ^13^C-labeled PEP was reduced by ~ 60% in the PEPCKi-treated cells (Additional file 4: Figure S4E). We also observed a decrease in ^13^C-labeled pyruvate, the downstream product of PEP (Additional file 4: Figure S4F). Although the decrease in pyruvate could be a result of pyruvate/OAA recycling, coupled with the decrease in PEP, these data demonstrate that PEPCKi is inhibiting PEPCK in colon-derived cancer cells.

### PEPCK inhibitor decreases growth

**Fig. 3:**
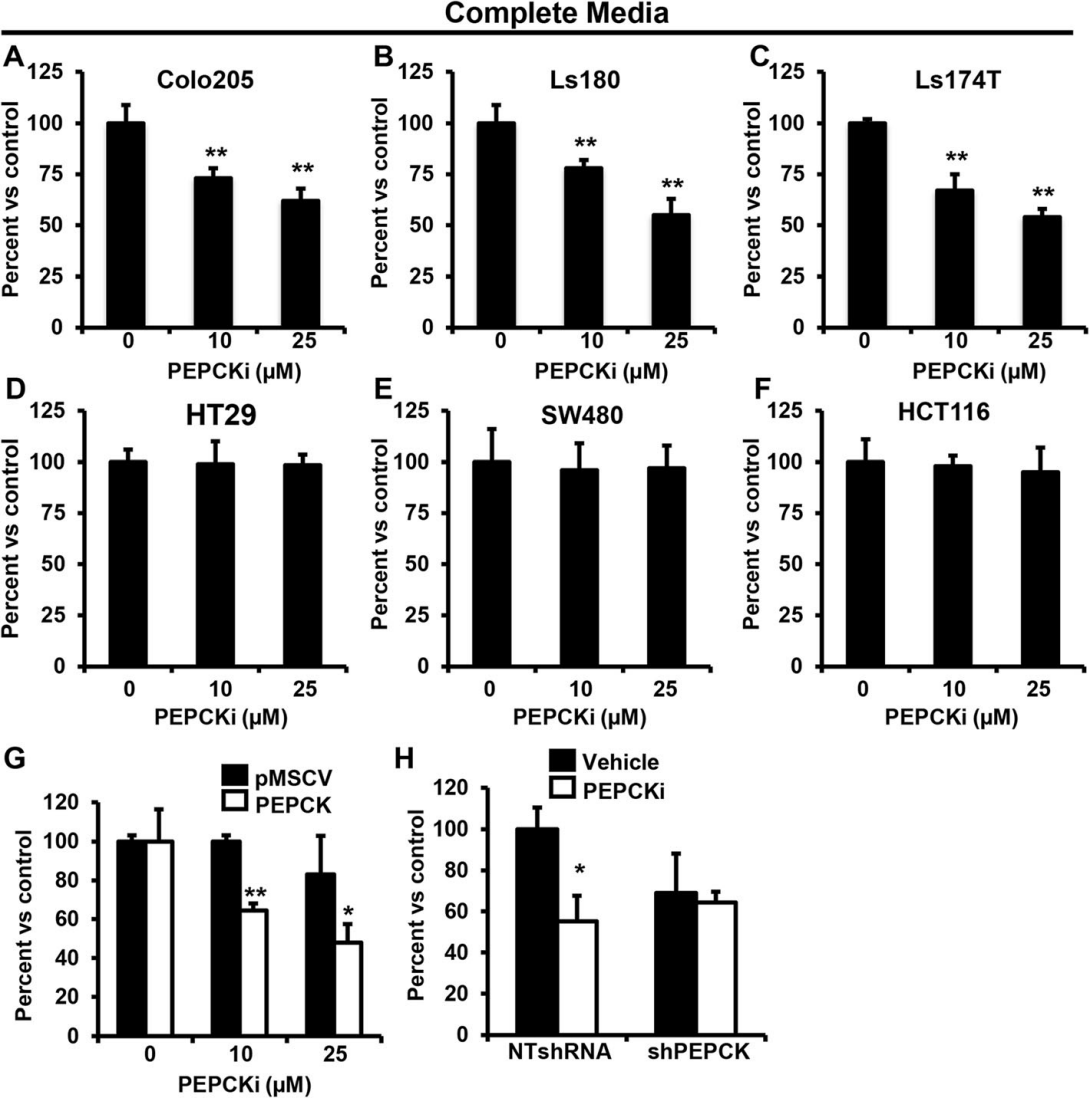
PEPCKi decreases proliferation in colorectal cancer cells. **a**–**f** Colo205, Ls180, Ls174T, HT29, SW180, and HCT116 cells, respectively, were treated with PEPCKi and cell number determined. **g** HT29 cells stably expressing an empty vector or PEPCK were treated with indicated doses of PEPCKi and cell number determined. **h** Colo205 cells with shNT or shPEPCK were treated 10 μM PEPCKi and cell number determined. Cells were seeded and treated with indicated doses of inhibitor for up to 5 days. *N* = 3 ± SD. *p < 0.05, **p < 0.01

Next, we examined the effect of the PEPCKi on cell growth. Initially, we used complete media and observed a dose-dependent decrease in cell number in several PEPCK expressing cell lines (Fig. 3a–c and Additional file 4: Figure S4G). In contrast, cells that do not express PEPCK were unaffected by similar doses of the PEPCKi (Fig. 3d–f and Additional file 4: Figure S4G). Indeed, the small rescue of growth by lactate in HT29 cells under reduced nutrient conditions might reflect this lack of PEPCK (Fig. 1d). To further confirm the specificity of the PEPCKi, we treated cells with genetic gain or loss of PEPCK, in which we had previously had shown that PEPCK promotes cell growth [20]. Treatment of HT29 vector control cells (lacking endogenous PEPCK) with the PEPCKi did not alter cell proliferation (Fig. 3g and Additional file 4: Figure S4H). However, in cells with ectopic expression of PEPCK, cell proliferation was reduced by ~ 50% following treatment with 25 μM PEPCKi. We also examined the effect of the PEPCKi in Colo205 cells with a non-target shRNA or shRNA against PEPCK [20]. The PEPCKi reduced cell proliferation in NT shRNA Colo205 cells, whereas the effect on proliferation was blunted in Colo205 cells with knockdown of PEPCK (Fig. 3h and Additional file 3: Figure S3A). These studies demonstrate that pharmacological inhibition of PEPCK reduces the growth of colon cancer cells expressing PEPCK in complete media.

**Fig. 4:**
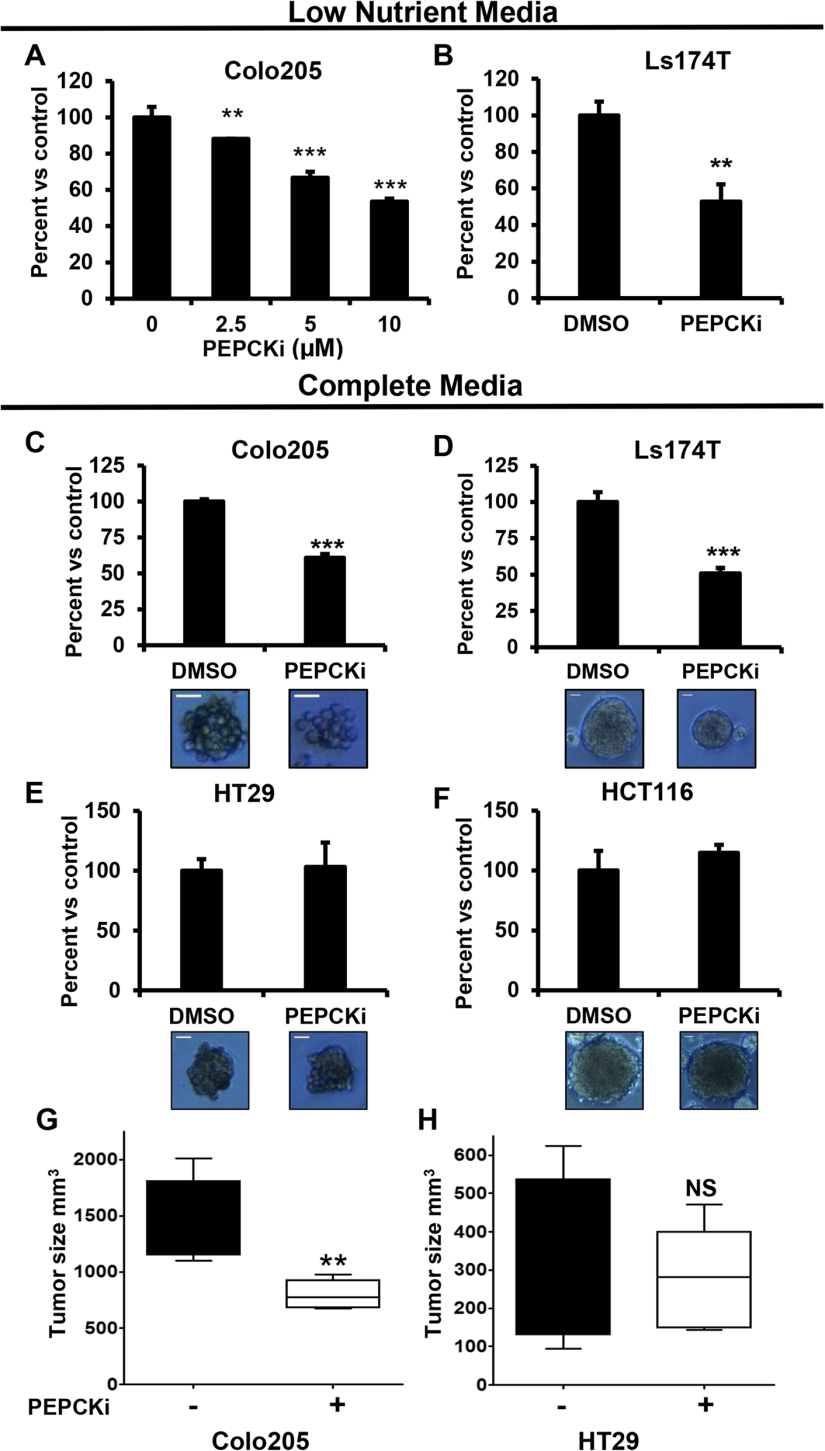
PEPCKi decreases tumor growth. **a** Colo205 and **b** LS174T cells, were treated with 0–10 μM PEPCKi in reduced nutrient media and cell number determined after 3 days. *N* = 3 ± S.D. **c** Colo205, **d** Ls174T, **e** HT29, and **f** HCT116 cells were grown as spheroids for 3 days, treated with 15 μM PEPCKi, and spheroid size was determined after 2 days using ImageJ. *N* = 3 ± S.D. Scale bar = 35 μm. **h** Colo205 and **i** HT29 cells were inoculated into the flank of 6-week-old nude mice. Once tumors became palpable, mice were administered 10 mg/kg PEPCK inhibitor every other day and tumors measured for an additional 10 days. *N* = 6 ± SD. NS Not significant, *p < 0.05, **p < 0.01, ***p < 0.001

Our initial data showed that lactate was most effective at restoring cell proliferation under reduced nutrient conditions. Therefore, we treated PEPCK expressing cells with the PEPCKi under more physiologically relevant conditions. Although reducing nutrient levels decreased growth of cells in general as described above, the PEPCKi inhibitor had an even greater effect on cell growth under these conditions. Twenty-five micromolars of the PEPCK inhibitor reduced growth by ~ 50% in complete media (Fig. 3a). Under reduced nutrient conditions, the same effect was achieved with ≤ 10 μM PEPCKi (Fig. 4a, b and Additional file 5: Figures S5A). Interestingly, while we did not observe effects of the PEPCK inhibitor on cell growth in Moser cells (which express less PEPCK than Colo205 cells), in complete media, under low-nutrient conditions, growth was significantly inhibited (Additional file 5: Figure S5A). Next, we wanted to confirm that the specificity of the PEPCKi under low-nutrient conditions. Colon cancer cells without PEPCK were treated with the PEPCKi under reduced nutrient conditions. Similar to the experiments in complete media, the PEPCKi did not alter cell number (Additional file 5: Figure S5B–C). This demonstrates not only specificity, but more importantly, that the PEPCKi is more effective at reducing cell growth under more physiologically relevant conditions.

We then wanted to determine if the effect on cell number was due to a decrease in proliferation. We treated cells in low-nutrient media with the PEPCKi and examined the expression of Ki67 using immunocytochemistry. The PEPCKi decreased Ki67 expression by 50% (Additional file 6: Figure S6A–B). FACS analysis revealed that treatment with the PEPCKi led to a significant decrease in the percentage of cells in G2 and an increase in the number of cells in S-phase (Additional file 6: Figure S6C). In addition, we did not observe evidence of apoptosis as determined by PARP cleavage or increased Annexin V staining (Additional file 6: Figure S6D–E). Taken together, these data indicate that the inhibitor decreases proliferation in part via effects on the cell cycle.

Tumor spheroids more accurately represent the tumor microenvironment [33]. Therefore, we cultured PEPCK expressing cells (Colo205 and LS174T) on low adherent plates to promote spheroid formation and then treated with the PEPCKi. Inhibition of PEPCK significantly reduced spheroid formation even in complete media (Fig. 4c, d). The effect of the PEPCKi on spheroid growth was similar in nutrient-deprived conditions to spheroids grown in complete media (Additional file 6: Figure S6F). Treatment of spheroids from cells that do not express PEPCK did not affect spheroid growth (Fig. 4e, f).

### PEPCKi decreases tumor growth in vivo

We subsequently used tumor xenografts to determine if the PEPCKi could alter tumor growth in vivo. Tumor xenografts were established, and then mice treated with the PEPCKi. PEPCKi treatment of mice with tumors expressing PEPCK (Colo205) reduced tumor growth ~ 50% (Fig. 4g). In contrast, treatment of mice with tumors that do not express PEPCK (HT29) did not reduce tumor growth (Fig. 4h). While systemic effects could in part explain the reduction in growth in the Colo205 tumors, the lack of an effect in mice with HT29 tumors suggests that it is most likely not an effect on systemic metabolism. These data demonstrate that the PEPCKi can inhibit tumor growth in vivo.

### PEPCKi induces metabolic stress

**Fig. 5:**
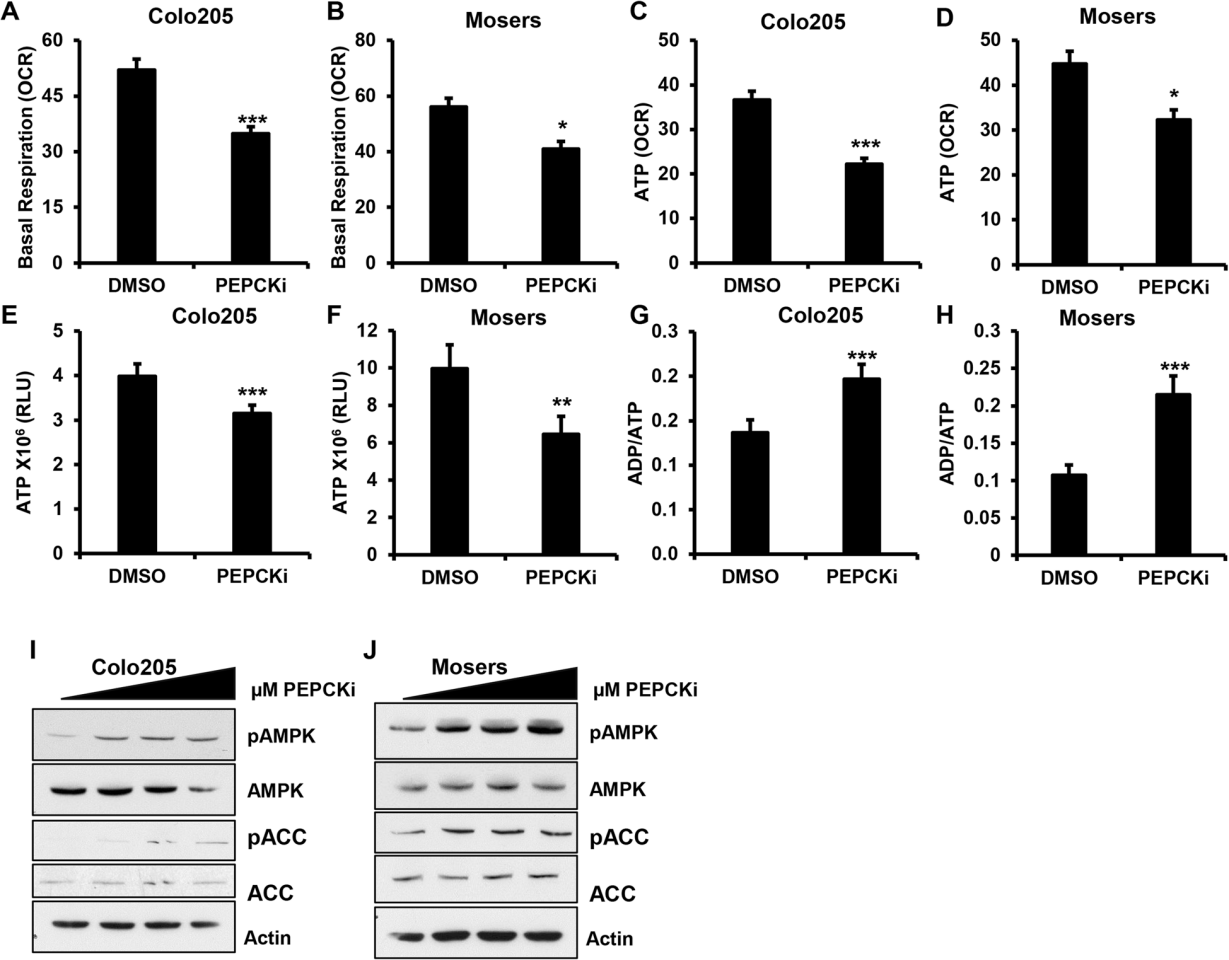
PEPCKi promotes bioenergetic stress. **a** Colo205 and **b** Moser cells were treated with 10 μM PEPCKi in reduced nutrient media and basal OCR measured using a XF^e^ Seahorse Bioanalyzer. **c** Colo205 and **d** Moser cells were treated with 10 μM PEPCKi in reduced nutrient media and ATP coupled respiration measured using a XF^e^ Seahorse Bioanalyzer. *N* ≥ 22 ± SEM. **e**–**h** Colo205 and Moser cells were treated with PEPCKi in low-nutrient conditions and ATP levels measured and ADP/ATP ratio determined. *N* ≥ 5 ± SD. **i**, **j** Colo205 and Moser cells were serum starved O/N and then treated with PEPCKi (0–10 μM) for 24 h and protein lysates analyzed via western blot *p < 0.05, **p < 0.01, ***p < 0.001

Next, we wanted to determine whether the PEPCKi altered the TCA cycle and OxPhos. We treated CRC cells with the PEPCKi overnight in reduced nutrient media and measured OCR. Interestingly, we found that basal respiration was decreased in cells treated with the PEPCKi in a dose-dependent manner indicating that the PEPCKi decreased OxPhos (Fig. 5a, b and Additional file 7: Figure S7A–B). The assay also revealed that the inhibitor decreased ATP levels as determined by ATP-coupled respiration (Fig. 5c, d and Additional file 7: Figure S7C). We further confirmed the decrease in ATP production using an enzyme-based assay. The PEPCKi decreased the levels of ATP with a corresponding increase in ADP/ATP ratio (Fig. 5e–h and Additional file 7: Figure S7D–E). This led us to examine the activation of AMPK, the cellular energy sensor, which is phosphorylated following bioenergetic stress. Treatment of cells with the PEPCKi led to an increase in AMPK phosphorylation (Fig. 5i, j and Additional file 7: Figure S7F). There was also an increase in phosphorylation of the AMPK target acetyl-CoA carboxylase. We do not see these effects in cells that do not express PEPCK (Additional file 7: Figure S7G). Collectively, these data suggest that inhibiting PEPCK blocks the utilization of substrates by the TCA cycle, disrupting cellular bioenergetics.

### Targeting lactate utilization with a PEPCK inhibitor

**Fig. 6:**
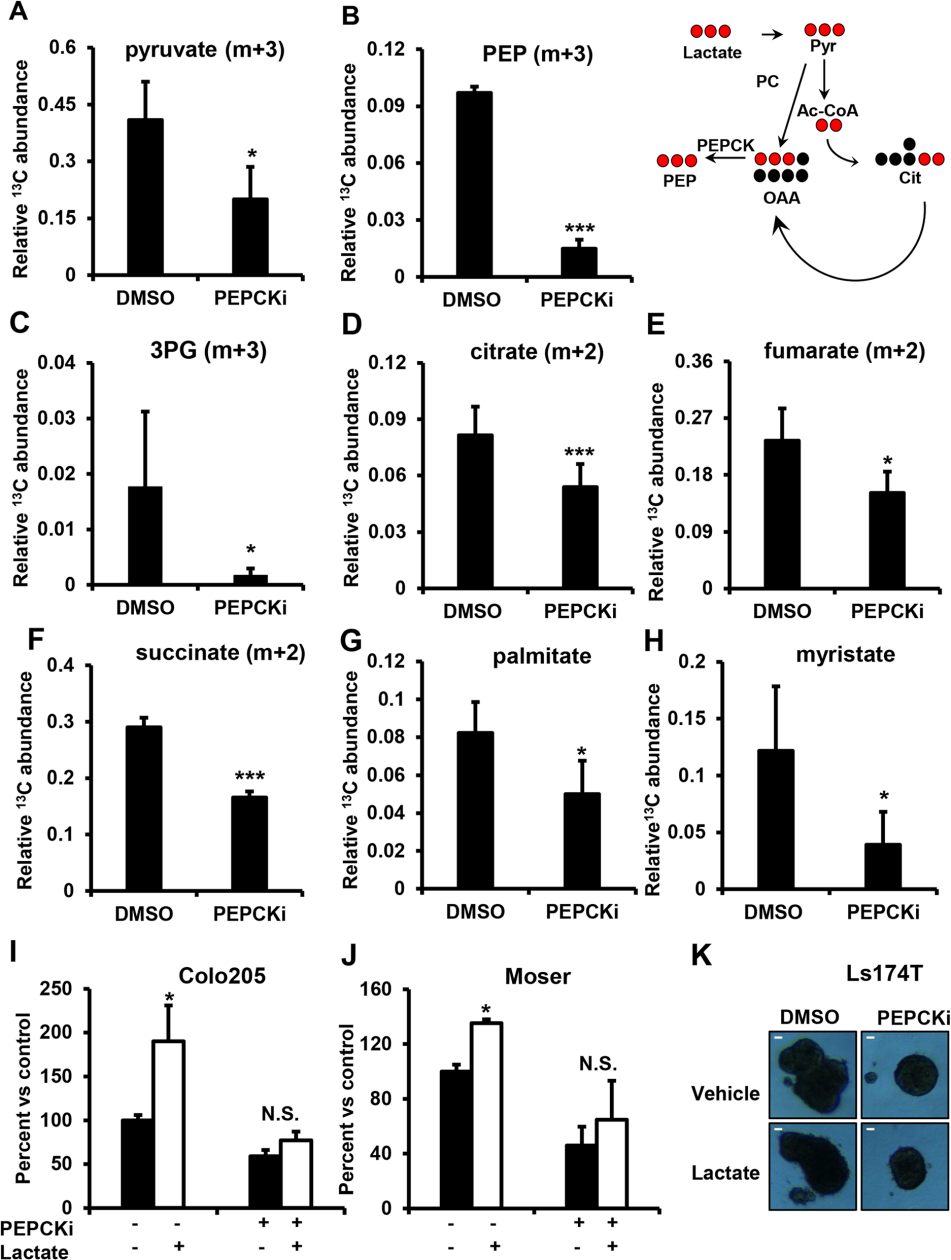
PEPCKi blocks lactate utilization. Relative abundance of m + 3 **a** pyruvate, **b** PEP, and **c** 3PG, and m + 2 **d** citrate, **e** fumarate, and **f** succinate were determined from Colo205 cells following incubation with ^13^C_3_ lactate and treated with PEPCKi. Relative abundance of **g** m + 16 palmitate and **h** m + 14 myristate were determined from Colo205 cells incubated with ^13^C_3_ lactate and treated with PEPCKi. Cells were incubated in reduced nutrient media with and without 10 μM PEPCKi overnight, incubated with 10 mM ^13^C lactate for 6 h (polar) or 16 h (non-polar), and metabolites harvested and analyzed using GC/MS. *N* ≥ 3 ± SD. **i** Colo205 and **j** Moser cells were treated with PEPCKi with and without lactate and cell number determined after 5 days. **k** Ls174T were grown as spheroids and treated with PEPCKi in the presence or absence of lactate. Images taken after 48 h. Spheroid size was measured using ImageJ. Scale bar = 50 μm. *N* = 3 ± SD. NS, not significant. *p < 0.05, **p < 0.01, ***p < 0.001

Our initial studies showed that colon cancer cells utilize lactate as bioenergetic and biosynthetic substrate in part via the TCA cycle. We also showed previously and in these studies that inhibition of PEPCK (genetically or pharmacologically) decreases these functions [20]. Therefore, we wanted to determine whether the PEPCKi would reduce lactate utilization and its effects on cell growth. Cells were treated with PEPCKi and ^13^C_3_-labeled lactate. The PEPCKi blocked ^13^C incorporation into the glycolytic intermediates pyruvate, PEP, and 3PG (Fig. 6a–c and Additional file 8: Figure S8A–C). Interestingly, unlike our knockdown studies, we see a decrease in total intracellular ^13^C_3_-labeled lactate (Additional file 8: Figure S8D). This difference could potentially be due to differences between acute inhibition and stable knockdown where cells have adapted. In order to test if PEPCK is sufficient to promote uptake of ^13^C lactate entry into the cell, we transiently overexpressed PEPCK using adenovirus and measured the total intracellular ^13^C lactate. We observe that overexpression of PEPCK significantly increases lactate inside the cell suggesting that PEPCK promotes lactate uptake (Additional file 8: Figure S8E). Next, we measured whether PEPCKi affects entry of lactate into the TCA cycle. We found that incorporation of ^13^C from lactate into citrate, fumarate, and succinate were reduced following treatment with the PEPCKi (Fig. 6d–f and Additional file 8: Figure S8F–H). We then examined whether PEPCK inhibition blocked anabolic use of lactate. Similar to PEPCK knockdown studies, the PEPCKi decreased the incorporation of lactate into palmitate and myristate by ~ 65% (Fig. 6g, h). These studies show that pharmacological inhibition of PEPCK not only decreases cell growth but also blocks the ability of colon cancer cells to utilize lactate to fuel their increased bioenergetic and anabolic needs.

Since we observe that PEPCK promotes lactate uptake (Additional file 8: Figure S8D–E), we sought to determine whether decreased utilization of lactate for metabolic intermediates was due to a decrease in uptake. To test this, colo205 cells were incubated with ^13^C_3_ lactate for 1 h prior to treatment with PEPCKi for 2 h. Cells treated with PEPCKi had similar levels of intracellular m + 3 lactate, demonstrating that short-term treatment with PEPCKi does not alter lactate uptake (Additional file 8: Figure S8I). Interestingly, we found that there was a decrease in the ^13^C incorporation into the TCA cycle intermediates, citrate and malate (Additional file 8: Figures S8J–K). In addition, there was less ^13^C incorporation into the glycolytic intermediate pyruvate and PEP (Additional file 8: Figure S8L–M). These data demonstrate that PEPCKi decreases metabolism of lactate and suggests that the decrease in ^13^C lactate uptake observed is due to reduced utilization.

Next, we wanted to determine whether the PEPCKi inhibitor could block the ability of CRC cells to utilize lactate to fuel their growth. Lactate increased the growth of cells in reduced nutrient media as expected ~ 2-fold (Fig. 6i, j). PEPCKi alone decreased cell proliferation > 40%. However, the ability of lactate to increase cell proliferation was completely blocked by the PEPCKi (Fig. 6i, j). We observed a similar effect genetically using shRNA against PEPCK (Additional file 9: Figure S9A). We also examined the effect of the PEPCKi and lactate in spheroids expressing PEPCK. Treatment of spheroids with the PEPCKi decreased spheroid size by ~ 50% in both the presence and absence of lactate (Fig. 6k). These data strongly suggest that PEPCK is in part responsible for the ability of cancer cells to utilize lactate as a substrate for growth.

## Discussion

Metabolic flexibility provides cancer cells with important growth advantages that enable them to grow depending on nutrient availability in the tumor microenvironment. Cancer cells use non-carbohydrate sources for bioenergetics pathways such as the TCA cycle and biosynthetic pathways including lipid and nucleic acid synthesis [1, 11, 20, 23]. Recent studies show that some cancers use lactate as a substrate for the TCA cycle to an even greater degree than glucose [17, 19, 24]. However, the mechanism regulating this effect is not clear. In this study, we show that CRC cells use lactate to fuel the TCA cycle for proliferation. Here, we demonstrate using genetic and pharmacologic approaches that PEPCK plays a role in the ability of CRC cells to utilize lactate for energy and anabolic metabolism.

Lactate levels (5–10 mM) in the TME are higher than glucose (0.1–0.5 mM) especially in hypoxic regions [13–16, 34]. Therefore, we cultured CRC cells with physiologically relevant concentrations of lactate in the presence of reduced glucose and glutamine concentrations. Lactate rescued growth inhibition of cells cultured in reduced nutrient media, which was associated with increased bioenergetics and utilization of lactate by the TCA cycle. The ability of lactate to maintain bioenergetics and the TCA cycle was blocked following inhibition of PEPCK. Inhibiting PEPCK decreased oxidative phosphorylation and subsequently, ATP production, leading to bioenergetic stress. AMPK is a key nutrient that becomes activated under nutrient stress. Treatment of cells with the PEPCKi increased AMPK phosphorylation. We also observed decreased Ki67 staining and S-phase cycle arrest. This is consistent with previous studies showing that nutrient deprivation can promote S-phase arrest [35, 36]. S-phase arrest in response to nutrient deprivation is also associated with an apoptosis-independent cell death [37–39]. These data supports an ability of PEPCK inhibition to activate AMPK and inhibit cell cycle progression by blocking nutrient utilization by cells.

Our studies show that PEPCK promotes lactate utilization via the TCA cycle. However, we also observe a role for lactate in biosynthetic pathways. Lactate is converted to pyruvate and then acetyl CoA which then condenses with OAA to form citrate. While citrate can be oxidized by the TCA cycle for bioenergetic purposes, citrate can also leave the mitochondria and be used as precursor for fatty acid synthesis, an important pathway in cancer [18]. We show that CRC incorporate lactate-derived ^13^C carbons into palmitate and myristate. This effect is reduced in PEPCK knockdown cells and following treatment with the PEPCKi, supporting our previous work showing that PEPCK promotes de novo fatty acid synthesis. Although previous studies demonstrate the incorporation of lactate into lipids using NMR, these are the first studies to show the use of lactate for fatty acid synthesis [19]. Those studies examined total lipid content, which encompasses many different lipid classes. It should be noted that while we did not perform detailed steady state isotopic studies, this concern is mitigated by the mass isotopomer data enrichment data with respect to incorporation of ^13^C into various metabolites. Our data further elucidates the importance of lactate in cancer cell growth and importantly illustrates its use for anabolic growth.

Our data demonstrates the PEPCK reduces incorporation of ^13^C lactate into the TCA cycle. We also show that there is decreased uptake of lactate with pharmacologic inhibition of PEPCK. However, this does not occur in knockdown cells, which do demonstrate reduced utilization, but not uptake. We propose that since cells cannot utilize lactate, they reduce uptake of lactate in response to inhibition of PEPCK. This is a phenomenon similar to what we previously described with glucose and glutamine [20]. Although we do demonstrate that PEPCK is sufficient to promote lactate uptake (Additional file 8: Figure S8E) and that PEPCKi alters lactate utilization in the absence of decreased lactate uptake (Additional file 8: Figure S8I–M), future studies should further elaborate on the effect of PEPCK on lactate uptake versus utilization.

Previous studies reported that the mitochondrial isoform of PEPCK, PCK2, promotes lactate incorporation into PEP in lung cancer cells enabling cells to survive glucose depletion [20, 21]. Those studies showed that an inhibitor of PEPCK, 3 mercaptopicolinic acid (3MPA), blocks incorporation of lactate into PEP. 3MPA was discovered to inhibit PEPCK over 60 years ago. However, the effects of 3MPA require millimolar concentrations, which are difficult to achieve clinically. In contrast, we observed effects of the PEPCKi using micromolar concentrations of PEPCKi. In addition, the utilization of lactate in colon cancer, especially with regard to PEPCK1 and lactate incorporation into the TCA cycle and anabolic precursor, have not been previously described.

## Conclusions

Utilization of lactate for glucose production via gluconeogenesis, its primary role in the liver, requires PEPCK and the TCA cycle (Fig. 6b) [40]. Recent studies shows the importance of lactate for fueling the TCA cycle of cancer cells highlighting the need to target lactate utilization. Until recently, most studies focused on targeting lactate production or transport [22]. No studies have focused on inhibiting lactate utilization of cancer cells via the TCA cycle. Our studies demonstrate that inhibition of PEPCK decreases lactate utilization to fuel the TCA cycle, anabolic metabolism, and cell proliferation, offering a novel therapeutic avenue.

## Additional files

Additional file 1: **Figure S1.** Related to Fig. 1. Colon-derived cancer cells use lactate for growth. (A–D) Colo205, Ls174T, Moser, and HT29 cells, respectively, were cultured with and without 10 mM lactate and cell number determined after 6 days using a Countess automated cell counter. *N* = 3 ± S.D. (E) Colo205 cells were cultured in complete or reduced nutrient media and cell number determined after 6 days. (F) Colo205 cells were cultured in the presence of increasing doses of lactate and cell number determined after 6 days *N* = 3 ± S.D *p < 0.05, **p < 0.01, ***p < 0.001. (DOCX 175 kb)

Additional file 2: **Figure S2.** Related to Fig. 1. Colon-derived cancer cells use lactate to fuel the TCA cycle. (A–C) Colo205, Ls174T, and Moser cells, respectively, were cultured in reduced nutrient media with and without 10 mM lactate and extracellular acidification rate (ECAR) measured using a XF^e^ Seahorse Bioanalyzer. *N* ≥ 15 ± SEM. (D) Colo205 cells were cultured in reduced nutrient media with ^13^C lactate for 6 h and total percent enrichment of ^13^C into the TCA cycle was measured (middle). Isotopologue distribution of ^13^C lactate enrichment of TCA cycle (surrounding). (E) Colo205 cells were cultured with ^13^C lactate in high glucose media and fractional percent enrichment of ^13^C into the TCA cycle was measured. *N* ≥ 3 ± SD *p < 0.05, **p < 0.01, ***p < 0.001. (DOCX 237 kb)

Additional file 3: **Figure S3.** Related to Fig. 2. PEPCKi decreases growth in colorectal cancer cells (A) PEPCK expression from Colo205 cells with shNT or shPEPCK analyzed via western blot. (B) intracellular m + 3 lactate relative abundance were determined in shNT or shPEPCK colo205 cells following incubation with ^13^C_3_ lactate. (C) Percent ^12^C and ^13^C enrichment of palmitate from colo205 cells incubated with ^13^C lactate *N* ≥ 3 ± SD. (DOCX 91 kb)

Additional file 4: **Figure S4.** Related to Fig. 3. PEPCKi decreases growth in colorectal cancer cells. (A) 3-alkyl-1,8-dibenzylxanthine (PEPCKi). (B) OAA and (C) PEP from Colo205 cells treated with PEPCKi were measured using an in vitro assay *N* = 3 ± SD. (D) Schematic for conversion of ^13^C_5_ glutamine into various metabolites. (E–F) Relative abundance of ^13^C. (E) PEP and (F) pyruvate was determined from colo205 cells treated with 25 μM PEPCKi and cultured with 4 mM ^13^C_5_ glutamine for 16 h and analyzed using GCMS *N* ≥ 3 ± S.D. *p < 0.05. (G) Protein expression of PEPCK from various colon cancer cell lines analyzed by western blot. (H) Protein expression of PEPCK in HT29 colon cancer cells that were stably infected with PEPCK or pmscv control analyzed by western blot. (DOCX 277 kb)

Additional file 5: **Figure S5.** Related to Fig. 3. PEPCKi decreases growth in colorectal cancer in vivo. (A–D) Ls174T, Moser, HCT116, and HT29 cells, respectively, were cultured in low-nutrient conditions, treated with PEPCKi and cell number determined after 3 days. *N* = 3 ± SD *p < 0.05, **p < 0.01, ***p < 0.001. (DOCX 134 kb)

Additional file 6: **Figure S6.** Related to Fig. 3. PEPCKi decreases proliferation. (A–B) Colo205 cells were treated with the PEPCKi and Ki67 expression analyzed using a confocal microscope. Values were quantified using ImageJ. *N* = 3 ± S.D. (C) Colo205 cells were treated with PEPCKi, stained with PI and analyzed by flow cytometry. Data is averaged over three independent experiments *N* = 3 ± SEM. (D) Colo205 cells were treated with PEPCKi and percent apoptosis was determined. Cells were stained with Annexin V and 7AAD and analyzed by flow cytometry. *N* = 3 ± S.D. (E) Colo205 cells were treated with PEPCKi and PARP cleavage analyzed via western blot. (F) Ls174T cells were grown as spheroids in reduced nutrient media, treated with 15 μM PEPCKi and spheroid size determined after 2 days using ImageJ. Scale bar = 50 μm *p < 0.05, **p < 0.01, ***p < 0.001. (DOCX 584 kb)

Additional file 7: **Figure S7.** Related to Fig. 5. PEPCKi induces metabolic stress. (A–B) Colo205 and Ls174T cells were treated with PEPCKi and basal respiration determined. (C) Ls174T cells were treated with PEPCKi and ATP levels measured *N* ≥ 22 ± SEM. (D–E) Ls174T cells were treated with PEPCKi in low-nutrient conditions and ATP levels measured and ADP/ATP ratio determined. ATP and ADP were measured using a luminescence assay. *N* ≥ 5 ± SD. (F–G) Ls174T and HCT116 cells were treated with PEPCKi (0–10 μM) for 24 h and analyzed via western blot *p < 0.05, **p < 0.01, ***p < 0.001. (DOCX 266 kb)

Additional file 8: **Figure S8.** Related to Fig. 6. PEPCKi blocks lactate utilization. Isotopalogue distribution of (A) pyruvate, (B) PEP, (C) 3PG, and (D) lactate relative abundance were determined from Colo205 cells incubated with ^13^C_3_ lactate with and without PEPCKi and analyzed using GCMS. (E) m + 3 lactate relative abundance was determined from HCT116 cells incubated with ^13^C_3_ lactate with and without PEPCK and analyzed using GCMS (top). Western blot of HCT116 with PEPCK overexpression using adenovirus (bottom). Isotopalogue distribution of (F) citrate, (G) fumarate and (H) succinate relative abundance were determined from Colo205 cells incubated with ^13^C_3_ lactate with and without PEPCKi and analyzed using GCMS. m + 3 (I) lactate, (L) pyruvate, and (M) PEP and m + 4 (J) malate and (K) citrate relative abundance were determined from Colo205 cells incubated with ^13^C_3_ lactate prior to treatment with and without PEPCKi and analyzed using GCMS *N* ≥ 3 ± S.D. *p < 0.05, **p < 0.01, ***p < 0.001. (DOCX 495 kb)

Additional file 9: **Figure S9.** Related to Fig. 6. PEPCKi blocks lactate-induced growth. (A) Colo205 cells with shNT or shPEPCK were treated with PEPCKi with and without lactate and cell number determined after 5 days. *N* = 3 ± S.D. N.S. Not significant. *p < 0.05. (DOCX 44 kb)

## Data Availability

The datasets used and/or analyzed during the current study are available from the corresponding author on reasonable request.

## Abbreviations

3MPA: 3 Mercaptopicolinic acid
3PG: 3-Phosphoglycerate
7AAD: 7-Aminoactinomycin D
AMPK: AMP-activated protein kinase
ANOVA: Analysis of variance
ATCC: American Type Culture Collection
CoA: Coenzyme A
CRC: Colorectal cancer
DMEM: Dulbecco’s modified Eagle’s medium
DMSO: Dimethyl sulfoxide
ECAR: Extracellular acidification rate
ECL: Enhanced chemiluminescence
GNG: Gluconeogenesis
HRP: Horseradish peroxidase
OAA: Oxaloacetate
OCR: Oxygen consumption rate
OxPhos: Oxidative phosphorylation
PARP: Poly ADP ribose polymerase
PEP: Phosphoenolpyruvate
PEPCK: Phosphoenolpyruvate carboxykinase
PI: Propidium iodide
TME: Tumor microenvironment
TCA cycle: Tricarboxylic acid cycle

## References

[1] DeBerardinis RJ, Lum JJ, Hatzivassiliou G, Thompson CB (2008). The biology of cancer: metabolic reprogramming fuels cell growth and proliferation. Cell Metab..

[2] Cantor JR, Sabatini DM (2012). Cancer cell metabolism: one hallmark, many faces. Cancer Discov..

[3] Hanahan D, Weinberg RA (2011). Hallmarks of cancer: the next generation. Cell..

[4] Warburg O (1956). On the origin of cancer cells. Science..

[5] Dey R, Moraes CT (2000). Lack of oxidative phosphorylation and low mitochondrial membrane potential decrease susceptibility to apoptosis and do not modulate the protective effect of Bcl-x(L) in osteosarcoma cells. J Biol Chem..

[6] Pedersen PL (1978). Tumor mitochondria and the bioenergetics of cancer cells. Prog Exp Tumor Res..

[7] Ashton TM, McKenna WG, Kunz-Schughart LA, Higgins GS (2018). Oxidative phosphorylation as an emerging target in cancer therapy. Clin Cancer Res..

[8] Fantin VR, St-Pierre J, Leder P (2006). Attenuation of LDH-A expression uncovers a link between glycolysis, mitochondrial physiology, and tumor maintenance. Cancer Cell..

[9] Lim HY, Ho QS, Low J, Choolani M, Wong KP (2011). Respiratory competent mitochondria in human ovarian and peritoneal cancer. Mitochondrion..

[10] DeBerardinis RJ, Mancuso A, Daikhin E, Nissim I, Yudkoff M, Wehrli S (2007). Beyond aerobic glycolysis: transformed cells can engage in glutamine metabolism that exceeds the requirement for protein and nucleotide synthesis. Proc Natl Acad Sci U S A..

[11] Wise DR, Thompson CB (2010). Glutamine addiction: a new therapeutic target in cancer. Trends Biochem Sci..

[12] Vander Heiden MG, Cantley LC, Thompson CB (2009). Understanding the Warburg effect: the metabolic requirements of cell proliferation. Science..

[13] Hirayama A, Kami K, Sugimoto M, Sugawara M, Toki N, Onozuka H (2009). Quantitative metabolome profiling of colon and stomach cancer microenvironment by capillary electrophoresis time-of-flight mass spectrometry. Cancer Res..

[14] Kennedy KM, Scarbrough PM, Ribeiro A, Richardson R, Yuan H, Sonveaux P (2013). Catabolism of exogenous lactate reveals it as a legitimate metabolic substrate in breast cancer. PLoS One..

[15] Schroeder T, Yuan H, Viglianti BL, Peltz C, Asopa S, Vujaskovic Z (2005). Spatial heterogeneity and oxygen dependence of glucose consumption in R3230Ac and fibrosarcomas of the Fischer 344 rat. Cancer Res..

[16] Walenta S, Chau TV, Schroeder T, Lehr HA, Kunz-Schughart LA, Fuerst A (2003). Metabolic classification of human rectal adenocarcinomas: a novel guideline for clinical oncologists?. J Cancer Res Clin Oncol..

[17] Faubert B, Li KY, Cai L, Hensley CT, Kim J, Zacharias LG (2017). Lactate metabolism in human lung tumors. Cell..

[18] Hirschhaeuser F, Sattler UG, Mueller-Klieser W (2011). Lactate: a metabolic key player in cancer. Cancer Res..

[19] Hui S, Ghergurovich JM, Morscher RJ, Jang C, Teng X, Lu W (2017). Glucose feeds the TCA cycle via circulating lactate. Nature..

[20] Montal ED, Dewi R, Bhalla K, Ou L, Hwang BJ, Ropell AE (2015). PEPCK coordinates the regulation of central carbon metabolism to promote cancer cell growth. Mol Cell..

[21] Drori S, Girnun GD, Tou L, Szwaya JD, Mueller E, Xia K (2005). Hic-5 regulates an epithelial program mediated by PPARgamma. Genes Dev..

[22] Singh A, Ruiz C, Bhalla K, Haley JA, Li QK, Acquaah-Mensah G, et al. De novo lipogenesis represents a therapeutic target in mutant Kras non-small cell lung cancer. FASEB J. 2018:fj201800204. 10.1096/fj.201800204 PMC6219836 29906244

[23] Mullen AR, Wheaton WW, Jin ES, Chen PH, Sullivan LB, Cheng T (2011). Reductive carboxylation supports growth in tumour cells with defective mitochondria. Nature..

[24] Park S, Chang CY, Safi R, Liu X, Baldi R, Jasper JS (2016). ERRalpha-regulated lactate metabolism contributes to resistance to targeted therapies in breast cancer. Cell Rep..

[25] Sonveaux P, Vegran F, Schroeder T, Wergin MC, Verrax J, Rabbani ZN (2008). Targeting lactate-fueled respiration selectively kills hypoxic tumor cells in mice. J Clin Invest..

[26] Chaube B, Malvi P, Singh SV, Mohammad N, Meena AS, Bhat MK (2015). Targeting metabolic flexibility by simultaneously inhibiting respiratory complex I and lactate generation retards melanoma progression. Oncotarget..

[27] Corbet C, Bastien E, Draoui N, Doix B, Mignion L, Jordan BF (2018). Interruption of lactate uptake by inhibiting mitochondrial pyruvate transport unravels direct antitumor and radiosensitizing effects. Nat Commun..

[28] Sun Y, Liu S, Ferguson S, Wang L, Klepcyk P, Yun JS (2002). Phosphoenolpyruvate carboxykinase overexpression selectively attenuates insulin signaling and hepatic insulin sensitivity in transgenic mice. J Biol Chem..

[29] Gomez-Valades AG, Vidal-Alabro A, Molas M, Boada J, Bermudez J, Bartrons R (2006). Overcoming diabetes-induced hyperglycemia through inhibition of hepatic phosphoenolpyruvate carboxykinase (GTP) with RNAi. Mol Ther..

[30] He L, Sabet A, Djedjos S, Miller R, Sun X, Hussain MA (2009). Metformin and insulin suppress hepatic gluconeogenesis through phosphorylation of CREB binding protein. Cell..

[31] Foley LH, Wang P, Dunten P, Ramsey G, Gubler ML, Wertheimer SJ (2003). Modified 3-alkyl-1,8-dibenzylxanthines as GTP-competitive inhibitors of phosphoenolpyruvate carboxykinase. Bioorg Med Chem Lett..

[32] Foley LH, Wang P, Dunten P, Ramsey G, Gubler ML, Wertheimer SJ (2003). X-ray structures of two xanthine inhibitors bound to PEPCK and N-3 modifications of substituted 1,8-dibenzylxanthines. Bioorg Med Chem Lett..

[33] Pampaloni F, Reynaud EG, Stelzer EH (2007). The third dimension bridges the gap between cell culture and live tissue. Nat Rev Mol Cell Biol..

[34] Urasaki Y, Heath L, Xu CW (2012). Coupling of glucose deprivation with impaired histone H2B monoubiquitination in tumors. PLoS One..

[35] Tinnemans MM, Lenders MH, ten Velde GP, Blijham GH, Ramaekers FC, Schutte B (1995). S-phase arrest of nutrient deprived lung cancer cells. Cytometry..

[36] Patel D, Menon D, Bernfeld E, Mroz V, Kalan S, Loayza D (2016). Aspartate rescues S-phase arrest caused by suppression of glutamine utilization in KRas-driven cancer cells. J Biol Chem..

[37] Raina K, Agarwal C, Wadhwa R, Serkova NJ, Agarwal R (2013). Energy deprivation by silibinin in colorectal cancer cells: a double-edged sword targeting both apoptotic and autophagic machineries. Autophagy..

[38] Ellington AA, Berhow M, Singletary KW (2005). Induction of macroautophagy in human colon cancer cells by soybean B-group triterpenoid saponins. Carcinogenesis..

[39] Butler R, Mitchell SH, Tindall DJ, Young CY (2000). Nonapoptotic cell death associated with S-phase arrest of prostate cancer cells via the peroxisome proliferator-activated receptor gamma ligand, 15-deoxy-delta12,14-prostaglandin J2. Cell Growth Differ..

[40] Owen OE, Kalhan SC, Hanson RW (2002). The key role of anaplerosis and cataplerosis for citric acid cycle function. J Biol Chem..

