# Supplementary material for: Inhibition of phosphoenolpyruvate carboxykinase blocks lactate utilization and impairs tumor growth in colorectal cancer
Source: Cancer Metab. 2019 Aug 1;7:8. doi: 10.1186/s40170-019-0199-6 (PMC6670241; doi:10.1186/s40170-019-0199-6)
Supplement: Supplementary file 1 — Figure S1. Related to Fig. 1. Colon-derived cancer cells use lactate for growth. (A–D) Colo205, Ls174T, Moser, and HT29 cells, respectively, were cultured with and without 10 mM lactate and cell number determined after 6 days using a Countess automated cell counter. N = 3 ± S.D. (E) Colo205 cells were cultured in complete or reduced nutrient media and cell number determined after 6 days. (F) Colo205 cells were cultured in the presence of increasing doses of lactate and cell number determined after 6 days N = 3 ± S.D *p < 0.05, **p < 0.01, ***p < 0.001. (DOCX 175 kb) [file 40170_2019_199_MOESM1_ESM.docx]

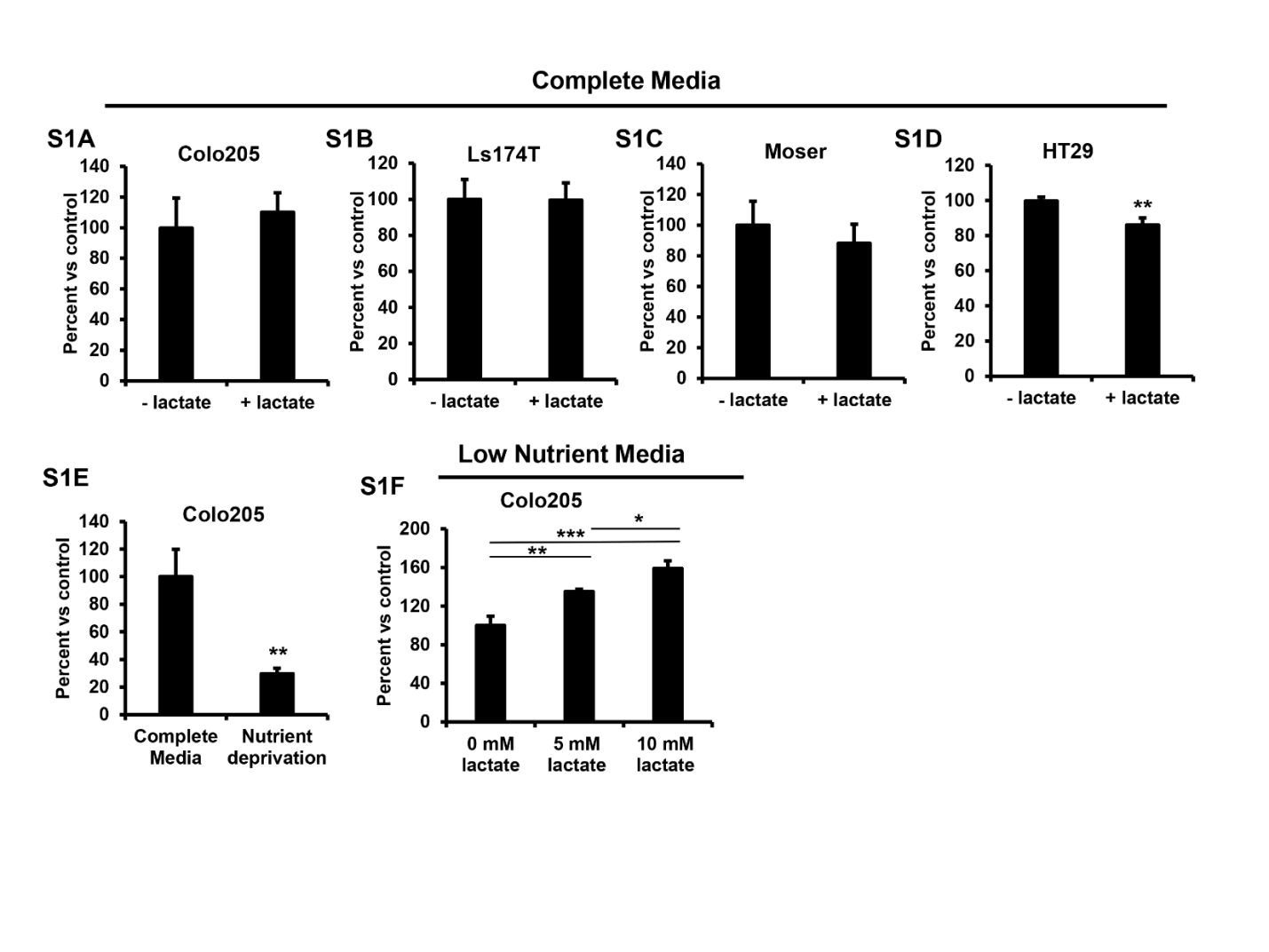
**Additional file 1 Figure S1. Related to Figure 1. Colon derived cancer cells use lactate for growth**. A-D) Colo205, Ls174T, Moser, and HT29 cells, respectively, were cultured with and without 10 mM lactate and cell number determined after 6 days using a Countess automated cell counter. N=3±S.D. E) Colo205 cells were cultured in complete or reduced nutrient media and cell number determined after 6 days. F) Colo205 cells were cultured in the presence of increasing doses of lactate and cell number determined after 6 days N=3±S.D * p<0.05, ** p<0.01, *** p<0.001
