# Supplementary material for: Inhibition of phosphoenolpyruvate carboxykinase blocks lactate utilization and impairs tumor growth in colorectal cancer
Source: Cancer Metab. 2019 Aug 1;7:8. doi: 10.1186/s40170-019-0199-6 (PMC6670241; doi:10.1186/s40170-019-0199-6)
Supplement: Supplementary file 2 — Figure S2. Related to Fig. 1. Colon-derived cancer cells use lactate to fuel the TCA cycle. (A–C) Colo205, Ls174T, and Moser cells, respectively, were cultured in reduced nutrient media with and without 10 mM lactate and extracellular acidification rate (ECAR) measured using a XFe Seahorse Bioanalyzer. N ≥ 15 ± SEM. (D) Colo205 cells were cultured in reduced nutrient media with 13C lactate for 6 h and total percent enrichment of 13C into the TCA cycle was measured (middle). Isotopologue distribution of 13C lactate enrichment of TCA cycle (surrounding). (E) Colo205 cells were cultured with 13C lactate in high glucose media and fractional percent enrichment of 13C into the TCA cycle was measured. N ≥ 3 ± SD *p < 0.05, **p < 0.01, ***p < 0.001. (DOCX 237 kb) [file 40170_2019_199_MOESM2_ESM.docx]

**
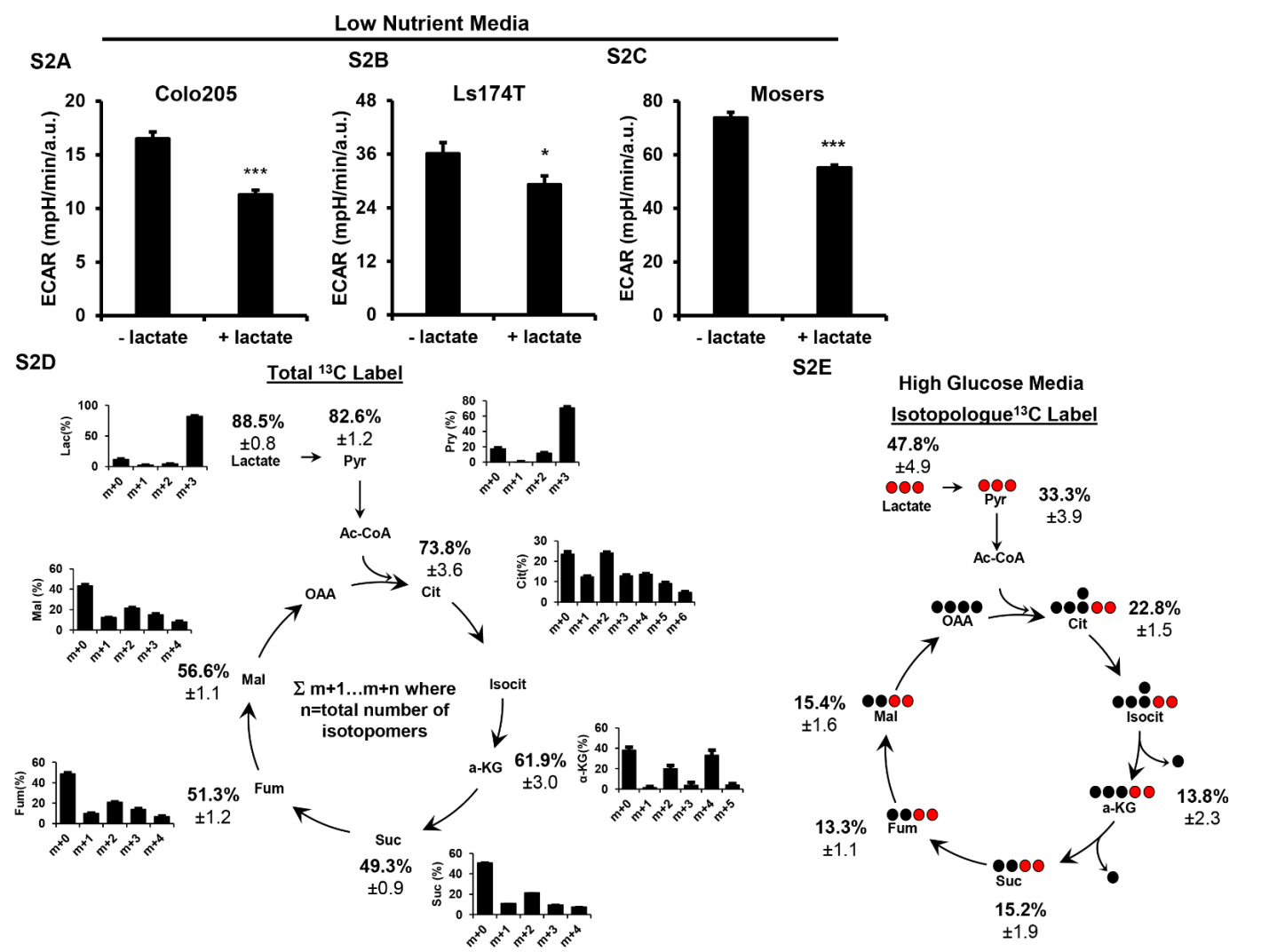
Additional file 2: Figure S2. Related to Figure 1. Colon derived cancer cells use lactate to fuel the TCA cycle** A-C) Colo205, Ls174T, and Moser cells, respectively, were cultured in reduced nutrient media with and without 10 mM lactate and extracellular acidification rate (ECAR) measured using a XF^e^ Seahorse Bioanalyzer. N≥15±SEM D) Colo205 cells were cultured in reduced nutrient media with ^13^C lactate for 6 hr and total percent enrichment of ^13^C into the TCA cycle was measured (middle). Isotopologue distribution of ^13^C lactate enrichment of TCA cycle (surrounding). E) Colo205 cells were cultured with ^13^C lactate in high glucose media and fractional percent enrichment of ^13^C into the TCA cycle was measured. N≥3± SD * p<0.05, ** p<0.01, *** p<0.001
