# Supplementary material for: Inhibition of phosphoenolpyruvate carboxykinase blocks lactate utilization and impairs tumor growth in colorectal cancer
Source: Cancer Metab. 2019 Aug 1;7:8. doi: 10.1186/s40170-019-0199-6 (PMC6670241; doi:10.1186/s40170-019-0199-6)
Supplement: Supplementary file 3 — Figure S3. Related to Fig. 2. PEPCKi decreases growth in colorectal cancer cells (A) PEPCK expression from Colo205 cells with shNT or shPEPCK analyzed via western blot. (B) intracellular m + 3 lactate relative abundance were determined in shNT or shPEPCK colo205 cells following incubation with 13C3 lactate. (C) Percent 12C and 13C enrichment of palmitate from colo205 cells incubated with 13C lactate N ≥ 3 ± SD. (DOCX 91 kb) [file 40170_2019_199_MOESM3_ESM.docx]

**
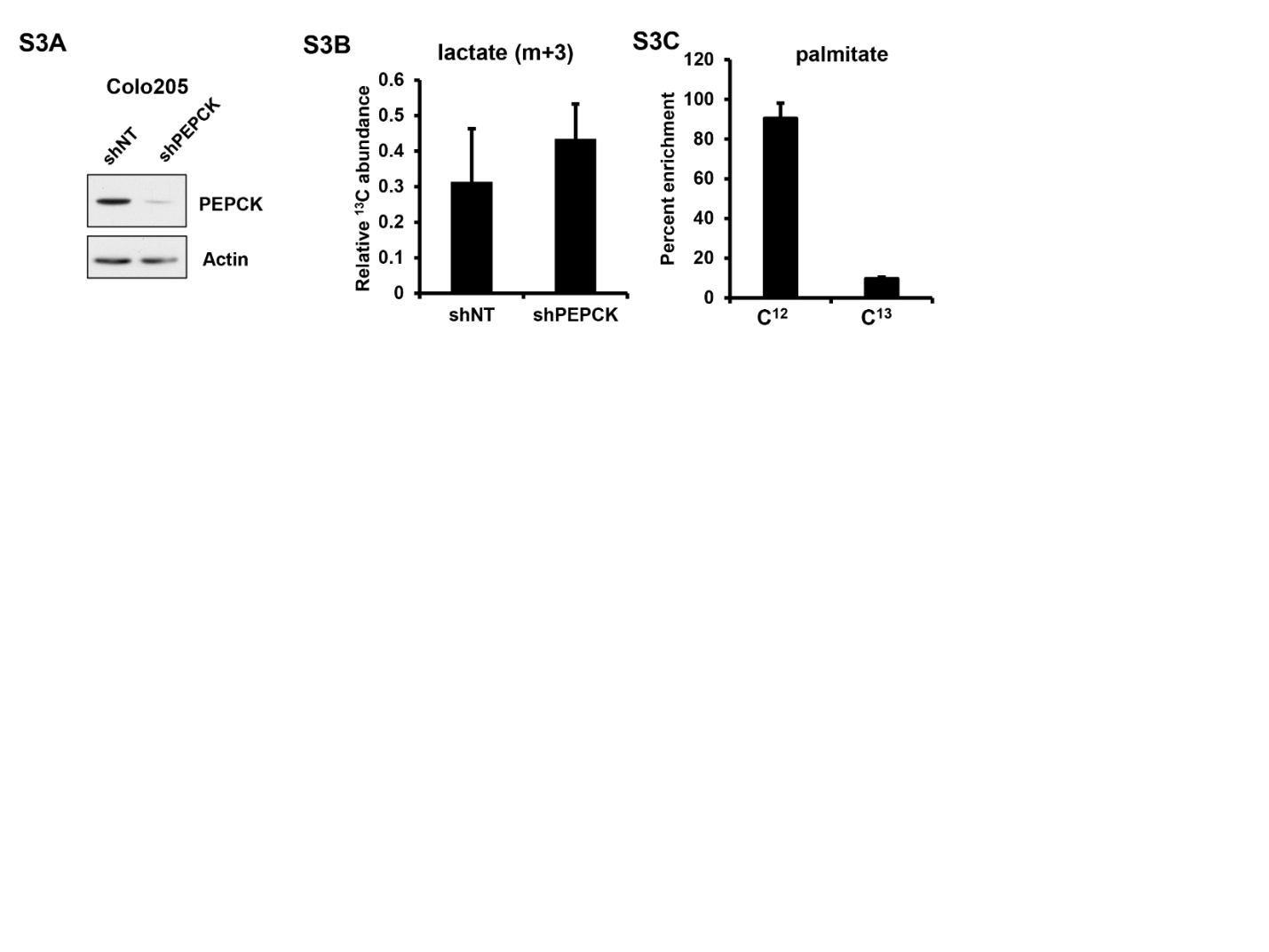
Additional file 3 Figure S3. Related to Figure 2. PEPCKi decreases growth in colorectal cancer cells** A) PEPCK expression from Colo205 cells with shNT or shPEPCK analyzed via western blot. B) intracellular m+3 lactate relative abundance were determined in shNT or shPEPCK colo205 cells following incubation with ^13^C_3_ lactate. C) Percent ^12^C and ^13^C enrichment of palmitate from colo205 cells incubated with ^13^C lactate N≥3± SD.
