# Supplementary material for: Inhibition of phosphoenolpyruvate carboxykinase blocks lactate utilization and impairs tumor growth in colorectal cancer
Source: Cancer Metab. 2019 Aug 1;7:8. doi: 10.1186/s40170-019-0199-6 (PMC6670241; doi:10.1186/s40170-019-0199-6)
Supplement: Supplementary file 4 — Figure S4. Related to Fig. 3. PEPCKi decreases growth in colorectal cancer cells. (A) 3-alkyl-1,8-dibenzylxanthine (PEPCKi). (B) OAA and (C) PEP from Colo205 cells treated with PEPCKi were measured using an in vitro assay N = 3 ± SD. (D) Schematic for conversion of 13C5 glutamine into various metabolites. (E–F) Relative abundance of 13C. (E) PEP and (F) pyruvate was determined from colo205 cells treated with 25 μM PEPCKi and cultured with 4 mM 13C5 glutamine for 16 h and analyzed using GCMS N ≥ 3 ± S.D. *p < 0.05. (G) Protein expression of PEPCK from various colon cancer cell lines analyzed by western blot. (H) Protein expression of PEPCK in HT29 colon cancer cells that were stably infected with PEPCK or pmscv control analyzed by western blot. (DOCX 277 kb) [file 40170_2019_199_MOESM4_ESM.docx]

**
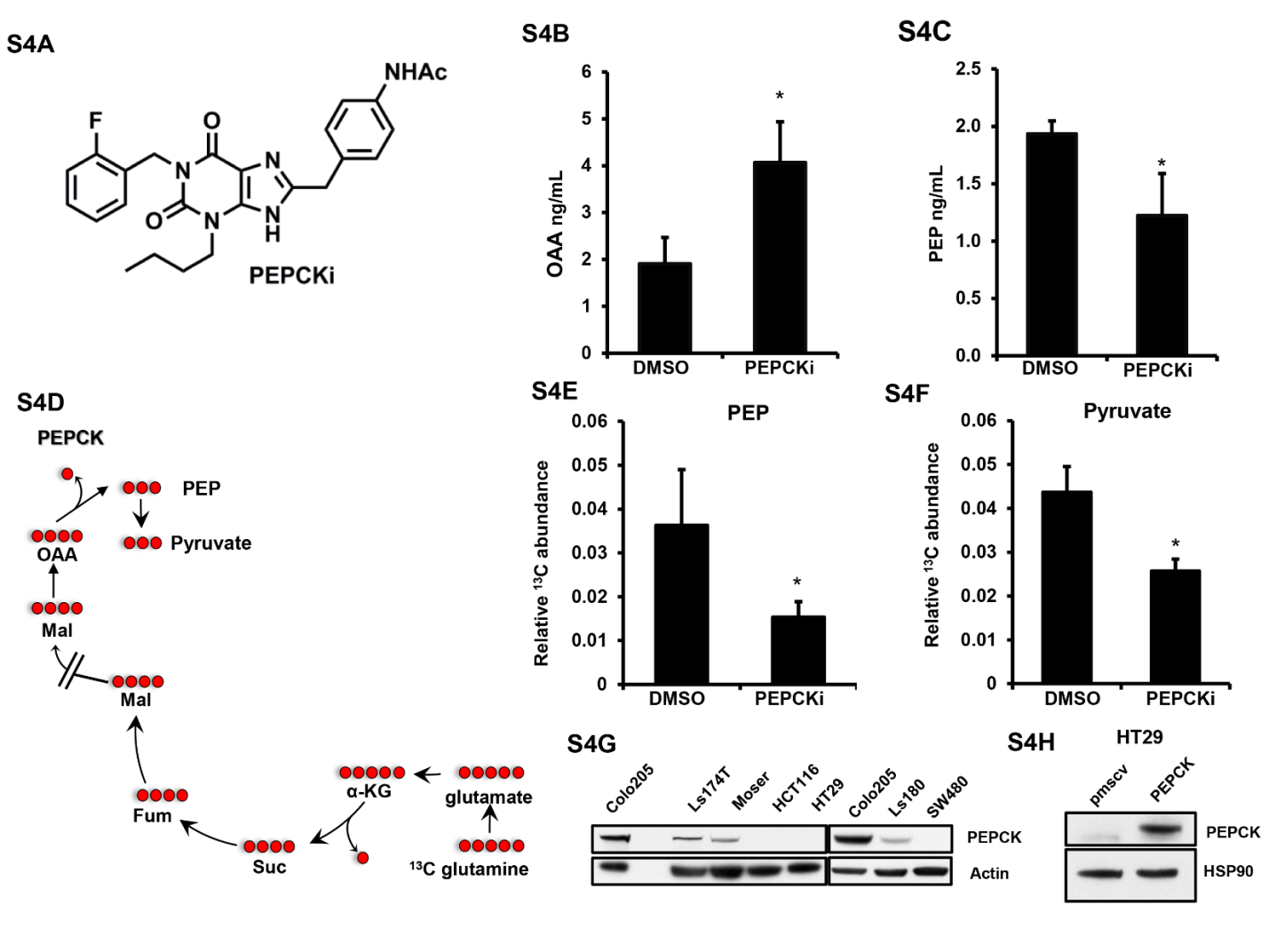
Additional file 4: Figure S4. Related to Figure 3. PEPCKi decreases growth in colorectal cancer cells.** A) 3-alkyl-1,8-dibenzylxanthine (PEPCKi). B) OAA and C) PEP from Colo205 cells treated with PEPCKi were measured using an *in vitro* assay N = 3 ± SD. D) Schematic for conversion of ^13^C_5_ glutamine into various metabolites. E-F) Relative abundance of ^13^C E) PEP and F) Pyruvate was determined from colo205 cells treated with 25 µM PEPCKi and cultured with 4mM ^13^C_5_ glutamine for 16 hours and analyzed using GCMS N≥3± S.D. * p < 0.05. G) Protein expression of PEPCK from various colon cancer cell lines analyzed by western blot. H) Protein expression of PEPCK in HT29 colon cancer cells that were stably infected with PEPCK or pmscv control analyzed by western blot.
