# Supplementary material for: Inhibition of phosphoenolpyruvate carboxykinase blocks lactate utilization and impairs tumor growth in colorectal cancer
Source: Cancer Metab. 2019 Aug 1;7:8. doi: 10.1186/s40170-019-0199-6 (PMC6670241; doi:10.1186/s40170-019-0199-6)
Supplement: Supplementary file 5 — Figure S5. Related to Fig. 3. PEPCKi decreases growth in colorectal cancer in vivo. (A–D) Ls174T, Moser, HCT116, and HT29 cells, respectively, were cultured in low-nutrient conditions, treated with PEPCKi and cell number determined after 3 days. N = 3 ± SD *p < 0.05, **p < 0.01, ***p < 0.001. (DOCX 134 kb) [file 40170_2019_199_MOESM5_ESM.docx]

**
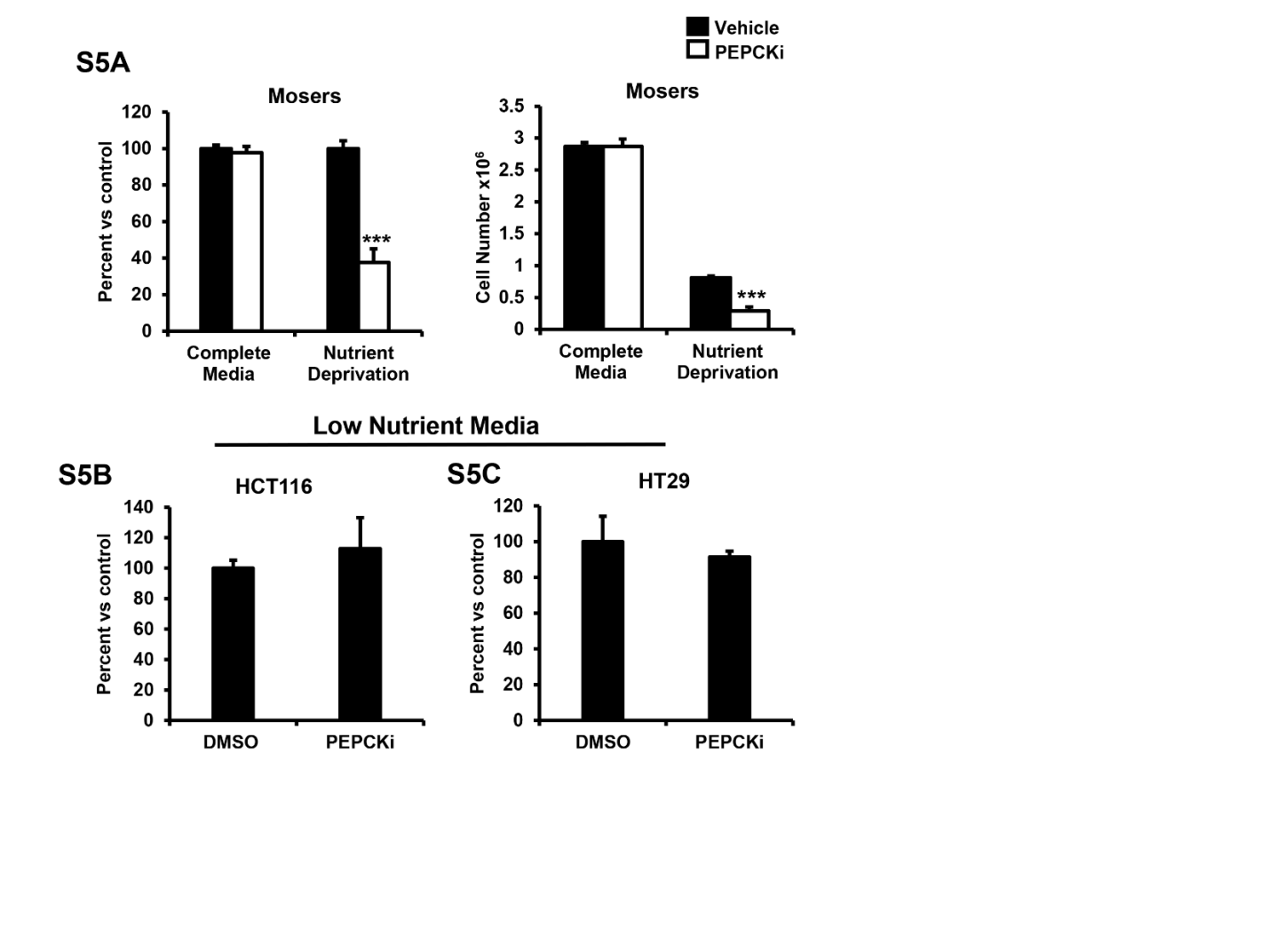
Additional file 5 Figure S5. Related to Figure 3. PEPCKi decreases growth in colorectal cancer in vivo.** A-D) Ls174T, Moser, HCT116, and HT29 cells, respectively, were cultured in low nutrient conditions, treated with PEPCKi and cell number determined after 3 days. N=3±SD * p<0.05, ** p<0.01, *** p<0.001
