# Supplementary material for: Inhibition of phosphoenolpyruvate carboxykinase blocks lactate utilization and impairs tumor growth in colorectal cancer
Source: Cancer Metab. 2019 Aug 1;7:8. doi: 10.1186/s40170-019-0199-6 (PMC6670241; doi:10.1186/s40170-019-0199-6)
Supplement: Supplementary file 6 — Figure S6. Related to Fig. 3. PEPCKi decreases proliferation. (A–B) Colo205 cells were treated with the PEPCKi and Ki67 expression analyzed using a confocal microscope. Values were quantified using ImageJ. N = 3 ± S.D. (C) Colo205 cells were treated with PEPCKi, stained with PI and analyzed by flow cytometry. Data is averaged over three independent experiments N = 3 ± SEM. (D) Colo205 cells were treated with PEPCKi and percent apoptosis was determined. Cells were stained with Annexin V and 7AAD and analyzed by flow cytometry. N = 3 ± S.D. (E) Colo205 cells were treated with PEPCKi and PARP cleavage analyzed via western blot. (F) Ls174T cells were grown as spheroids in reduced nutrient media, treated with 15 μM PEPCKi and spheroid size determined after 2 days using ImageJ. Scale bar = 50 μm *p < 0.05, **p < 0.01, ***p < 0.001. (DOCX 584 kb) [file 40170_2019_199_MOESM6_ESM.docx]

**
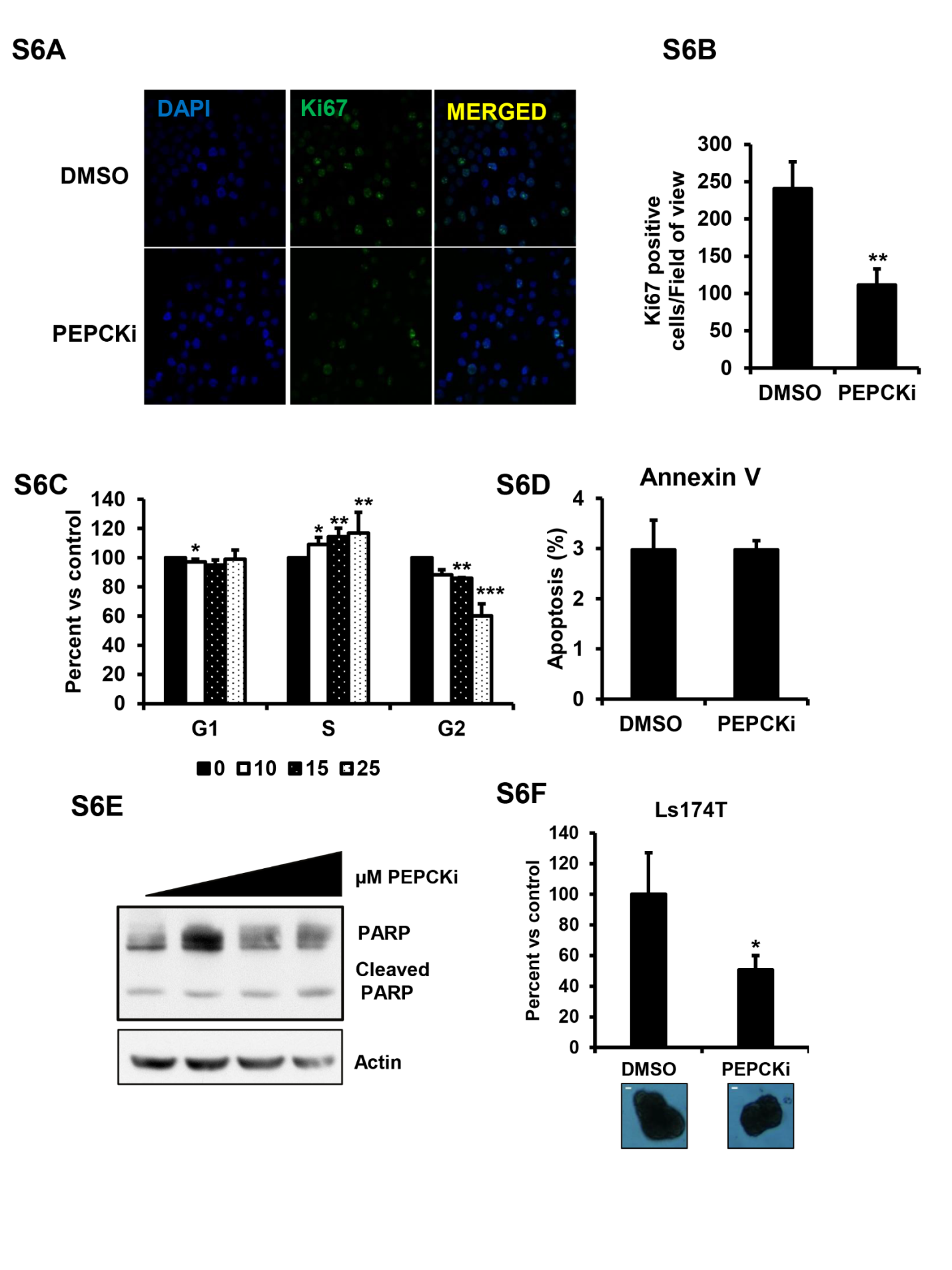
Additional file 6: Figure S6. Related to Figure 3. PEPCKi decreases proliferation** A-B) Colo205 cells were treated with the PEPCKi and Ki67 expression analyzed using a confocal microscope. Values were quantified using ImageJ. N=3±S.D. C) Colo205 cells were treated with PEPCKi, stained with PI and analyzed by flow cytometry. Data is averaged over 3 independent experiments N=3±SEM. D) Colo205 cells were treated with PEPCKi and percent apoptosis was determined. Cells were stained with Annexin V and 7AAD and analyzed by flow cytometry. N=3±S.D. E) Colo205 cells were treated with PEPCKi and PARP cleavage analyzed via western blot. F) Ls174T cells were grown as spheroids in reduced nutrient media, treated with 15 µM PEPCKi and spheroid size determined after 2 days using ImageJ. Scale bar = 50 µm * p<0.05, ** p<0.01, *** p<0.001
