# Supplementary material for: Inhibition of phosphoenolpyruvate carboxykinase blocks lactate utilization and impairs tumor growth in colorectal cancer
Source: Cancer Metab. 2019 Aug 1;7:8. doi: 10.1186/s40170-019-0199-6 (PMC6670241; doi:10.1186/s40170-019-0199-6)
Supplement: Supplementary file 7 — Figure S7. Related to Fig. 5. PEPCKi induces metabolic stress. (A–B) Colo205 and Ls174T cells were treated with PEPCKi and basal respiration determined. (C) Ls174T cells were treated with PEPCKi and ATP levels measured N ≥ 22 ± SEM. (D–E) Ls174T cells were treated with PEPCKi in low-nutrient conditions and ATP levels measured and ADP/ATP ratio determined. ATP and ADP were measured using a luminescence assay. N ≥ 5 ± SD. (F–G) Ls174T and HCT116 cells were treated with PEPCKi (0–10 μM) for 24 h and analyzed via western blot *p < 0.05, **p < 0.01, ***p < 0.001. (DOCX 266 kb) [file 40170_2019_199_MOESM7_ESM.docx]

**
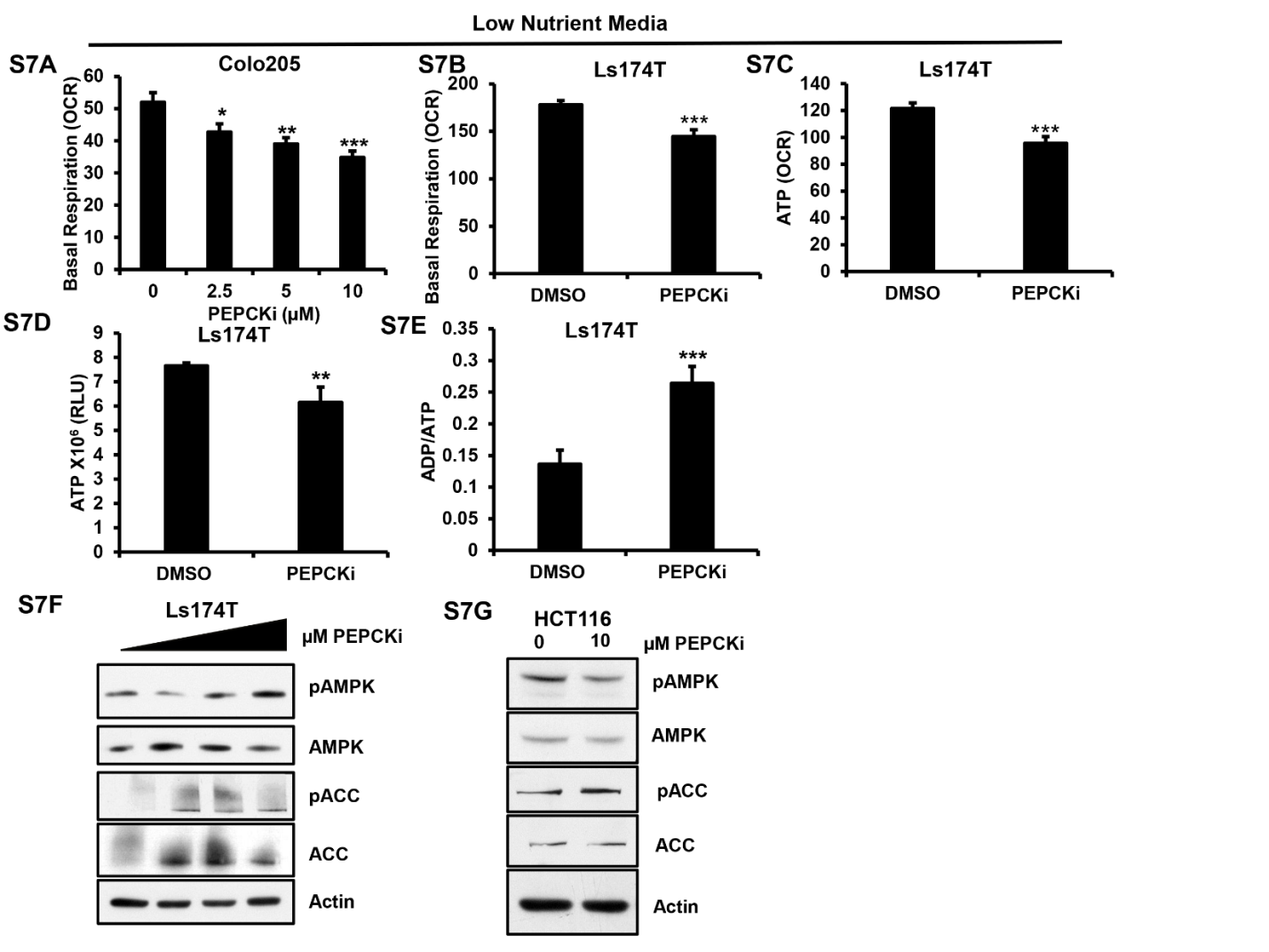
Additional file 7: Figure S7. Related to Figure 5. PEPCKi induces metabolic stress**. A-B) Colo205 and Ls174T cells were treated with PEPCKi and basal respiration determined. C) Ls174T cells were treated with PEPCKi and ATP levels measured N≥22±SEM. D-E) Ls174T cells were treated with PEPCKi in low nutrient conditions and ATP levels measured and ADP/ATP ratio determined. ATP and ADP were measured using a luminescence assay. N≥5±SD. F-G) Ls174T and HCT116 cells were treated with PEPCKi (0-10 µM) for 24 hrs and analyzed via western blot * p<0.05, ** p<0.01, *** p<0.001
