# Supplementary material for: Inhibition of phosphoenolpyruvate carboxykinase blocks lactate utilization and impairs tumor growth in colorectal cancer
Source: Cancer Metab. 2019 Aug 1;7:8. doi: 10.1186/s40170-019-0199-6 (PMC6670241; doi:10.1186/s40170-019-0199-6)
Supplement: Supplementary file 8 — Figure S8. Related to Fig. 6. PEPCKi blocks lactate utilization. Isotopalogue distribution of (A) pyruvate, (B) PEP, (C) 3PG, and (D) lactate relative abundance were determined from Colo205 cells incubated with 13C3 lactate with and without PEPCKi and analyzed using GCMS. (E) m + 3 lactate relative abundance was determined from HCT116 cells incubated with 13C3 lactate with and without PEPCK and analyzed using GCMS (top). Western blot of HCT116 with PEPCK overexpression using adenovirus (bottom). Isotopalogue distribution of (F) citrate, (G) fumarate and (H) succinate relative abundance were determined from Colo205 cells incubated with 13C3 lactate with and without PEPCKi and analyzed using GCMS. m + 3 (I) lactate, (L) pyruvate, and (M) PEP and m + 4 (J) malate and (K) citrate relative abundance were determined from Colo205 cells incubated with 13C3 lactate prior to treatment with and without PEPCKi and analyzed using GCMS N ≥ 3 ± S.D. *p < 0.05, **p < 0.01, ***p < 0.001. (DOCX 495 kb) [file 40170_2019_199_MOESM8_ESM.docx]

**
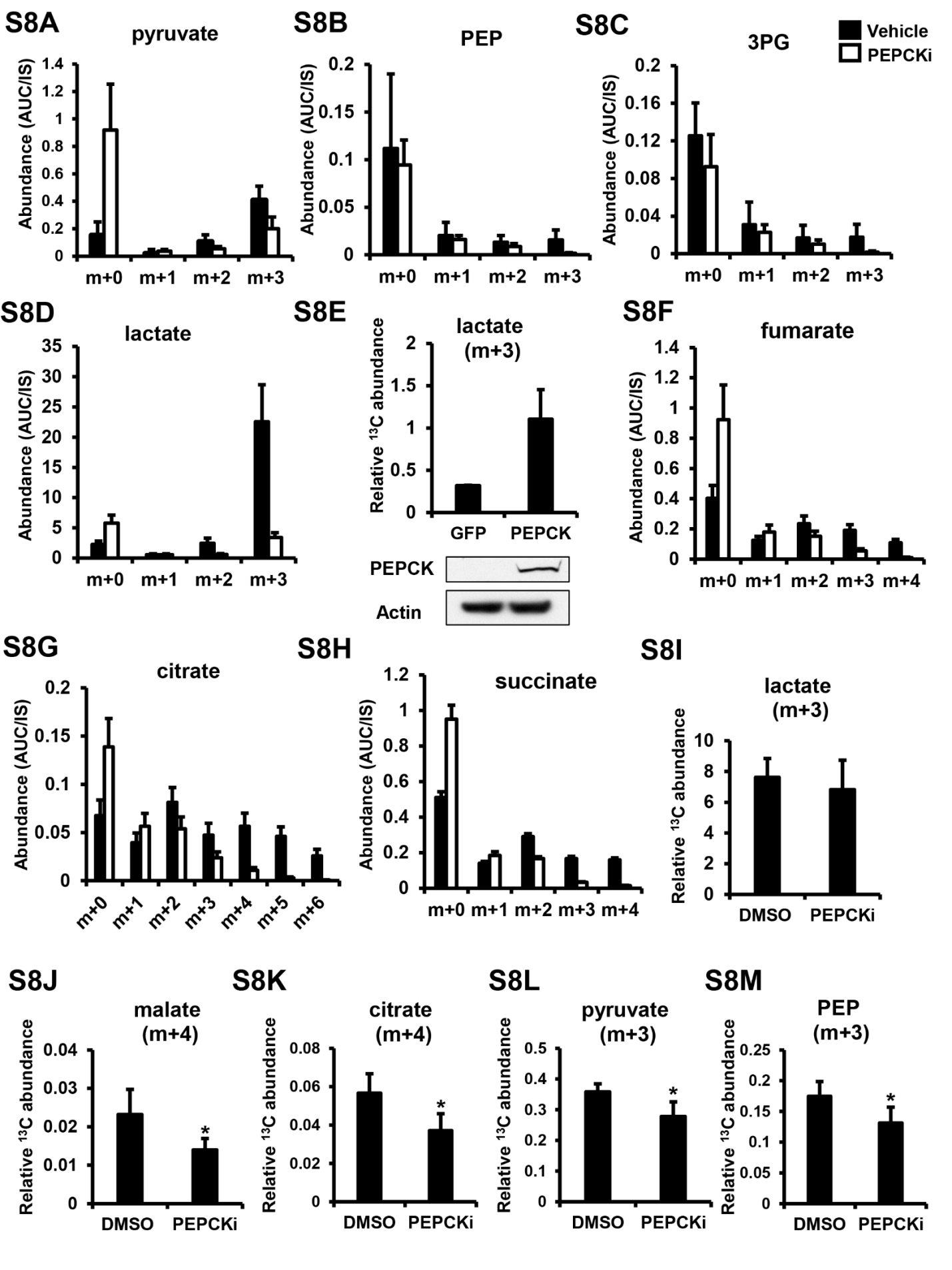
Additional file 8: Figure S8. Related to Figure 6. PEPCKi blocks lactate utilization**. Isotopalogue distribution of A) pyruvate, B) PEP, C) 3PG, D) and lactate relative abundance were determined from Colo205 cells incubated with ^13^C_3_ lactate with and without PEPCKi and analyzed using GCMS. E) m+3 lactate relative abundance was determined from HCT116 cells incubated with ^13^C_3_ lactate with and without PEPCK and analyzed using GCMS (top). Western blot of HCT116 with PEPCK overexpression using adenovirus (bottom). Isotopalogue distribution of F) citrate, G) fumarate and H) succinate relative abundance were determined from Colo205 cells incubated with ^13^C_3_ lactate with and without PEPCKi and analyzed using GCMS. m+3 I) lactate, L) pyruvate and M) PEP and m+4 J) malate and K) citrate relative abundance were determined from Colo205 cells incubated with ^13^C_3_ lactate prior to treatment with and without PEPCKi and analyzed using GCMS N≥3±S.D. * p<0.05, ** p<0.01, *** p<0.001.
