# Supplementary material for: Inhibition of phosphoenolpyruvate carboxykinase blocks lactate utilization and impairs tumor growth in colorectal cancer
Source: Cancer Metab. 2019 Aug 1;7:8. doi: 10.1186/s40170-019-0199-6 (PMC6670241; doi:10.1186/s40170-019-0199-6)
Supplement: Supplementary file 9 — Figure S9. Related to Fig. 6. PEPCKi blocks lactate-induced growth. (A) Colo205 cells with shNT or shPEPCK were treated with PEPCKi with and without lactate and cell number determined after 5 days. N = 3 ± S.D. N.S. Not significant. *p < 0.05. (DOCX 44 kb) [file 40170_2019_199_MOESM9_ESM.docx]

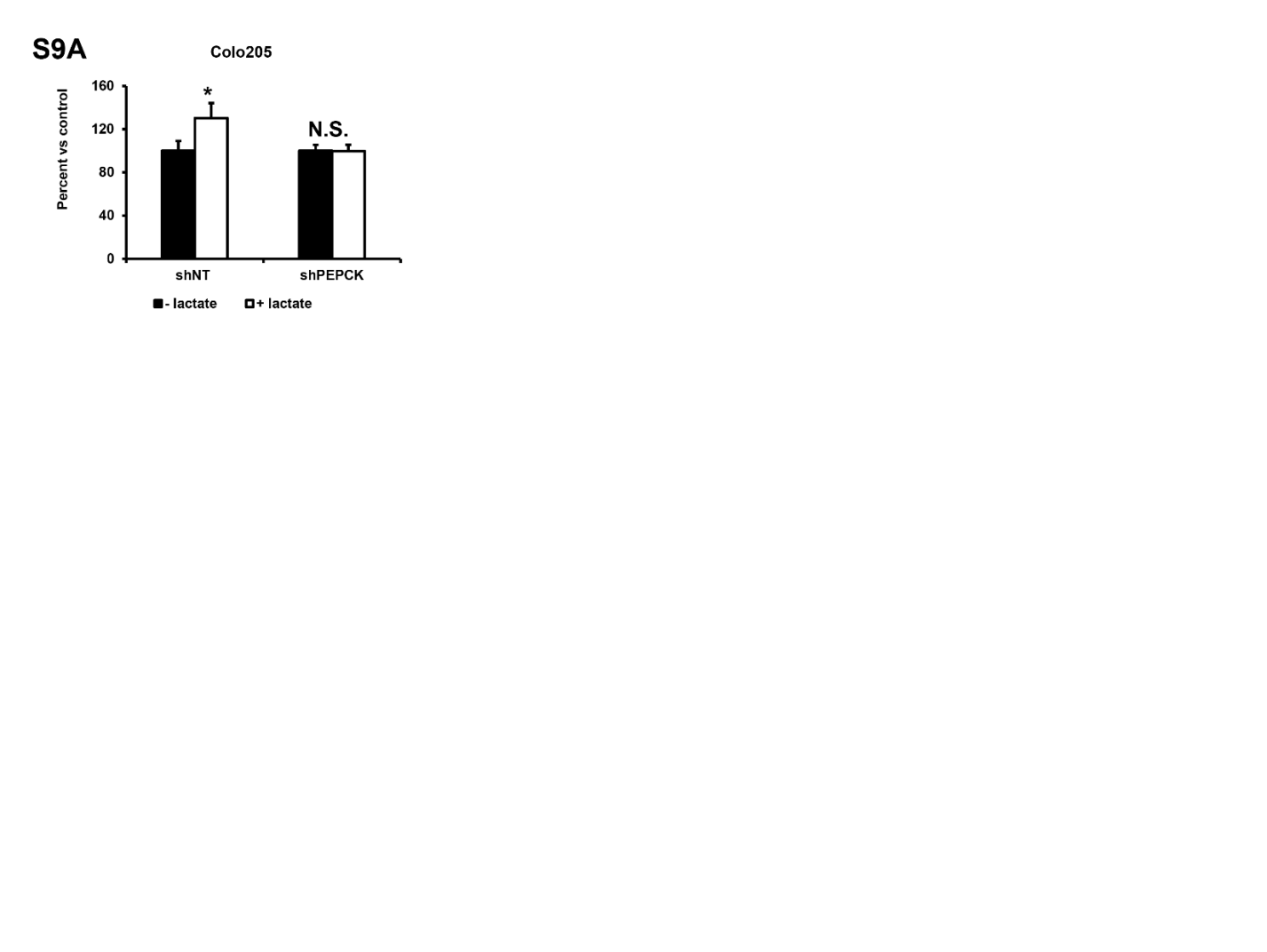
**Additional file 9: Figure S9. Related to Figure 6. PEPCKi blocks lactate induced growth.** A) Colo205 cells with shNT or shPEPCK were treated with PEPCKi with and without lactate and cell number determined after 5 days. N=3±S.D. N.S. Not significant. * p<0.05.
